# Dynamic finite-element simulations reveal early origin of complex human birth pattern

**DOI:** 10.1038/s42003-022-03321-z

**Published:** 2022-04-19

**Authors:** Pierre Frémondière, Lionel Thollon, François Marchal, Cinzia Fornai, Nicole M. Webb, Martin Haeusler

**Affiliations:** 1 UMR 7268 ADES, Aix Marseille University, EFS, CNRS, 51 boulevard Pierre Dramard, 13344 Marseille cedex 15, France; 2 Aix Marseille University, School of Midwifery, Faculty of Medical and Paramedical Sciences, 51 boulevard Pierre Dramard, 13344 Marseille cedex 15, France; 3 Aix Marseille University, UMR-T24, 51 boulevard Pierre Dramard, 13344 Marseille cedex 15, France; 4 Institute of Evolutionary Medicine, University of Zürich, Winterthurerstrasse 190, 8057 Zürich, Switzerland; 5 Department of Evolutionary Anthropology, University of Vienna, Djerassiplatz 1, 1030 Wien, Austria; 6 Human Evolution and Archaeological Sciences (HEAS), University of Vienna, Djerassiplatz 1, 1030 Wien, Austria; 7 Senckenberg Research Institute and Natural History Museum Frankfurt, Senckenberganlage 25, 60325, Frankfurt am Main, Germany; 8 Senckenberg Centre for Human Evolution and Palaeoenvironment, Institute of Archaeological Sciences, Eberhard Karls University of Tübingen, Rümelinstrasse 23, 72070 Tübingen, Germany; 9 Present Address: Vienna School of Interdisciplinary Dentistry—VieSID, Wasserzeile 35, 3400, Klosterneuburg, Austria

**Keywords:** Anthropology, Biological anthropology

## Abstract

Human infants are born neurologically immature, potentially owing to conflicting selection pressures between bipedal locomotion and encephalization as suggested by the obstetrical dilemma hypothesis. Australopithecines are ideal for investigating this trade-off, having a bipedally adapted pelvis, yet relatively small brains. Our finite-element birth simulations indicate that rotational birth cannot be inferred from bony morphology alone. Based on a range of pelvic reconstructions and fetal head sizes, our simulations further imply that australopithecines, like humans, gave birth to immature, secondary altricial newborns with head sizes smaller than those predicted for non-human primates of the same body size especially when soft tissue thickness is adequately approximated. We conclude that australopithecines required cooperative breeding to care for their secondary altricial infants. These prerequisites for advanced cognitive development therefore seem to have been corollary to skeletal adaptations for bipedal locomotion that preceded the appearance of the genus *Homo* and the increase in encephalization.

## Abstract (web summary)

Using finite element models of australopithecine pelvis reconstructions the secondary altricial nature of early hominin newborns is revealed, indicating that the need for cooperative breeding evolved secondary to bipedalism and thus prior to the appearance of the genus *Homo*.

## Introduction

During human birth, the fetus typically navigates a tight, convoluted birth canal by following a curved trajectory formed by the lumbar and sacral curves of the mother’s vertebral column, and typically needs to flex and rotate its head at various stages in order to successfully clear the bony pelvis^1^. This process is often hazardous for both the fetus and the mother and commonly requires the assistance of birth attendants, which contrasts to the generally faster and uncomplicated delivery of most other mammals^2,3^. Human neonates are born physically and neurologically more immature with smaller brains relative to those of adults compared with non-human primates. This adaptation is termed secondary altriciality as it represents a condition that is convergent in some aspects with altriciality, the ancestral life history trait retained in many mammals, e.g., in carnivores and many rodents^4^. However, in contrast to these species, human gestation length is not truncated and newborns are basically precocial like all other primates, being born as singletons with open eyes and ears, and displaying the adult pattern of hair coverage^4–7^. The emergence of these features of the human-like birth pattern has been attributed to opposing selection pressures related to encephalization and the changes facilitating biomechanical efficiency during bipedal locomotion, which has dramatically reshaped the pelvis over the course of hominin evolution^8^.

This alleged trade-off, known as the obstetrical dilemma, is notoriously difficult to test and has recently been challenged on multiple fronts^9–14^. Here, we employ an evolutionary approach focusing on australopithecines, the earliest known hominins with well-preserved pelvic remains, to explore the origins of the complex birth pattern characteristic of modern humans. While retaining relatively small brain sizes^15^, australopithecines already displayed morphological adaptations to bipedalism which included a shortened distance between the sacroiliac and hip joints similar to modern humans. This reduced torque during upright terrestrial bipedalism, but ultimately constrained the size of the birth canal. Analyses using australopithecines therefore permit differentiation between obstetrical adaptations explicitly related to bipedal locomotion from those related to our large brain size.

Previous attempts to reconstruct the evolution of human birth yielded conflicting results because of diverse estimates for fetal head size and different reconstructions of pelvic canal shape of the fossil hominins^16–22^ while the soft tissue lining of the birth canal was rarely taken into account^19^. Most studies used skull dimensions of a newborn chimpanzee as a proxy for newborn head size in australopithecines^16–18^ although these early hominins had a slightly larger mean adult brain volume than chimpanzees (420–459 cm^3^ compared with 369 cm^3^; ref. ^23^). Alternatively, estimates of newborn head size of australopithecines based on adult body size^24^ or adult brain size have been used^19,22^. Non-human primates, including great apes, have a neonatal brain size that is on average 43% of adult brain size, while this ratio is only 28% in modern humans^7^. It is controversial whether this different neonatal-to-adult brain size proportion can simply be explained by allometry^25^, or whether humans have a relatively smaller newborn brain size due to secondary altriciality^7^. Using the scaling relationship of neonatal-to-adult brain size based on 27 primate species^7^, mean neonatal brain mass for *Australopithecus afarensis, A. africanus* and *A. sediba* is estimated to a range of 166–184 g (Supplementary Table 1). In contrast, using the ratio typical of modern humans, a mean neonatal brain size of between 111 and 121 g is predicted for *Australopithecus*. On the other hand, a regression equation based on seven catarrhine primates including humans^25^ predicts neonatal brain sizes of 157–168 g that are slightly smaller than those inferred from the general primate formula.

**Fig. 1:**
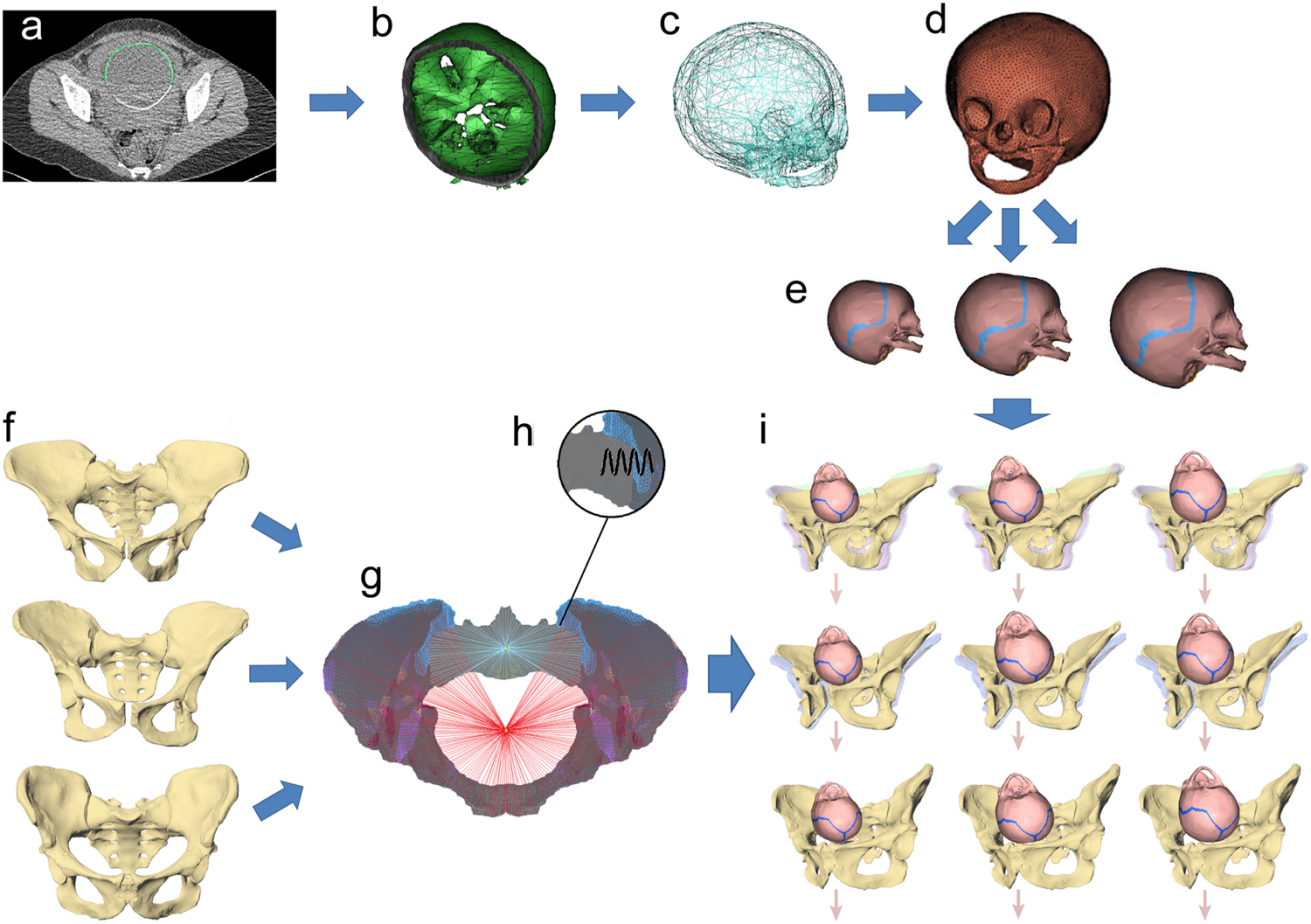
Workflow for modelling the australopithecine mother-infant dyads. **a** Segmentation of a modern human fetal skull out of the mother’s pelvic CT scan; **b**, **c** intermediate steps in the generation of the 3D model of the fetal head, showing the base of the skull and the compete skull; **d** re-meshing to generate shell elements and to apply the material proprieties of the fontanels and skull; **e** warping of the fetal skull based on three brain sizes: 110, 145, and 180 g; **f** pelvis reconstructions of A.L. 288-1, Sts 14 and MH2 (from top to bottom); **g** data setting for the pelvic meshes including assignment of material properties and boundary conditions; **h** representation of the sacro-iliac joint with a spring allowing nutation; **i** combination of skulls and pelvic meshes leading to a total of 21 mother-fetus dyads (corresponding to the four pelvic reconstructions for A.L. 288-1, two reconstructions of Sts 14, and one reconstruction of MH2), and application of gravity as the force of descent on the skull meshes. Multiple pelvic reconstructions of the same fossil are shown in different colours.

**Fig. 2:**
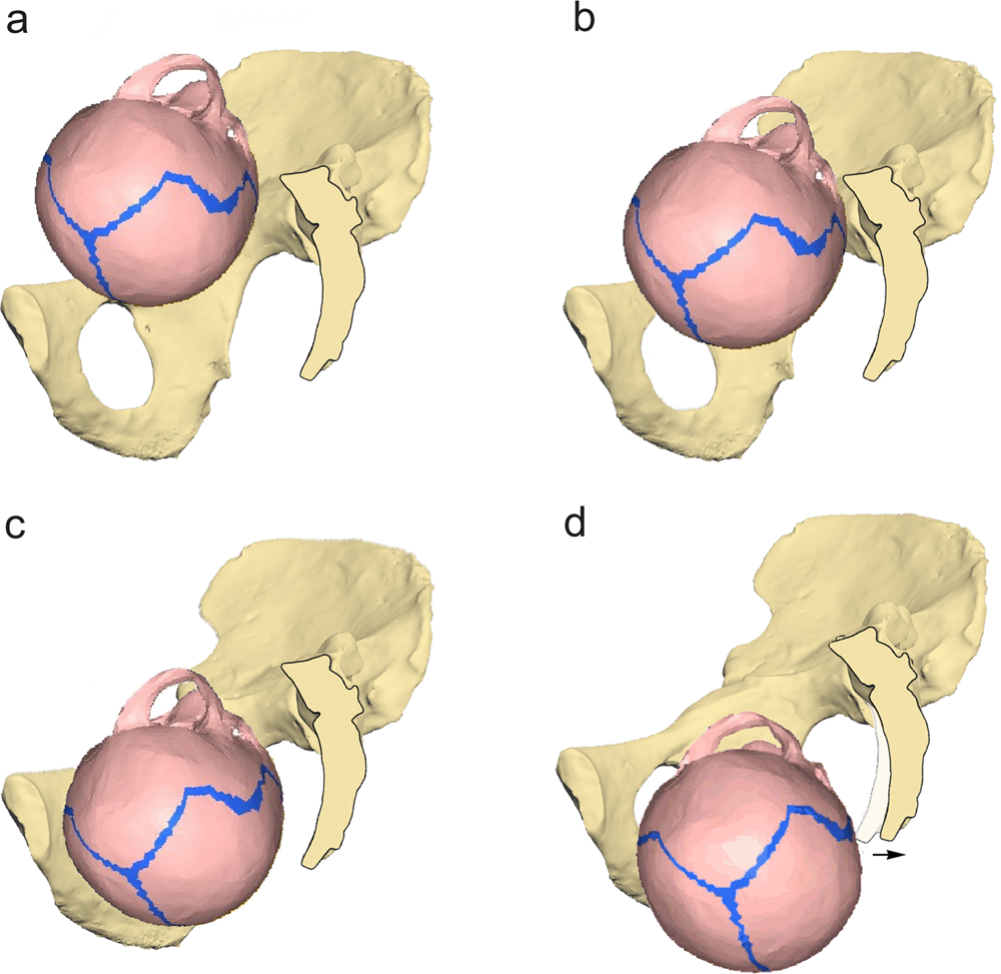
Phases of bony birth simulation. A fetal head with a 145 g brain size passes through the pelvis of Sts 14 as reconstructed by Häusler and Schmid^19^, depicted in lateral view. **a** Position of the fetal head at the onset of the simulation in left occiput anterior position; **b** engagement of the head at the pelvic inlet level; **c** descent through the pelvic midplane after slight rotation; note that internal rotation into the expected sagittal head orientation does not occur in this model due to the absence of soft tissue. **d** Expulsion from the outlet with backward nutation of the sacrum by 11 mm (arrow) and further rotation (45° in total) into a transverse orientation. Only the right hemipelvis is shown for visualization purposes.

For this study, we therefore scaled a fetal head model to three different neonatal brain sizes: 180 g, corresponding to a submaximal brain size using a general primate neonatal-to-adult brain size ratio; 110 g, which is close to the minimum predicted brain size using a modern human ratio; and an intermediate value of 145 g (Fig. 1). To account for the complex shape of the birth canal, the dynamic nature of childbirth, and the unique reaction forces resulting from fetopelvic contact, we performed dynamic 3D finite-element simulations of the birth process in australopithecines (Fig. 2) considering all published pelvic reconstructions. Given the heart-shaped pelvic inlet of australopithecines, which closely resembles the modern human shape, we started our simulations with the fetal head in a left occiput anterior (LOA) orientation as it is characteristic of modern humans (see Methods). The descent was then modeled using the force of gravity and the resultant interactions of the fetal head with the mother’s pelvis. Specifically, iterative calculations were performed considering different degrees of fetal head rotations and flexion. We also simulated sacro-iliac joint laxity, which typically occurs in all primates including humans^26^, whereas the pubic symphysis was kept immobile similar to great apes and modern humans^27,28^. The resulting cephalopelvic gap between the bony pelvic canal and the fetal skull was then compared with values for fetopelvic soft-tissue thickness derived from human intrapartum MRI scans and sonographic measurements (see Methods). The suitability of the bony pelvic shape for predicting fetal head rotation was assessed with a birth simulation based on a modern human pelvis.

We found that only our 145 g and the 110 g fetal head sizes successfully passed through the bony pelvic inlet and midplane. However, after accounting for adequate soft tissue space, only the 110 g fetal head size led to an eutocic birth. This implies that australopithecines had significantly smaller newborn brain sizes than predicted for a general primate. The rotational behaviour observed in our bony finite-element birth simulation of an average modern human also challenges previous assumptions that early hominins maintained a transverse head orientation at each pelvic plane during birth^18,22^. Australopithecines therefore seem to have likely evolved a more human-like birth pattern with secondary altriciality prior to the appearance of substantial encephalization characterizing the genus *Homo*, which is in line with the predictions of the obstetrical dilemma hypothesis^6,8^.

## Results and discussion

### Bony finite element analysis (FEA) simulations

**Fig. 3:**
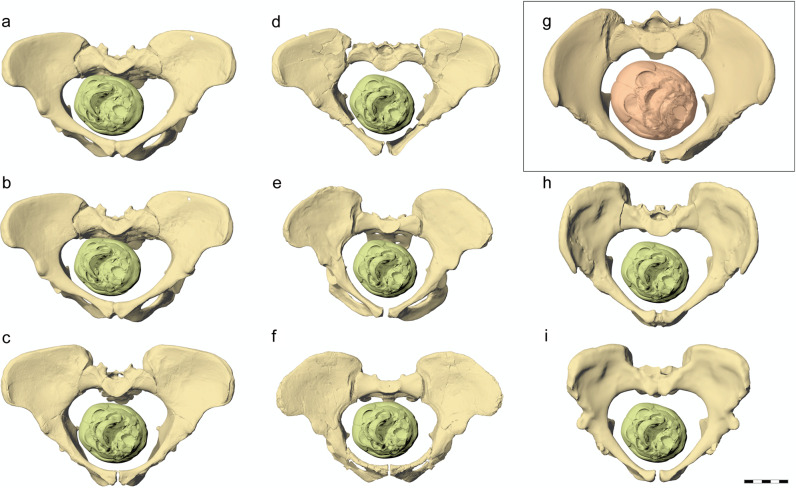
Australopithecine pelvic reconstructions with a 110 g fetal head size engaged in the pelvic inlet, including A.L. 288-1 (*Australopithecus afarensis*), Sts 14 (*A. africanus*), and MH2 (*A. sediba*), compared with an average sized modern human female pelvis and fetal head. A.L. 288-1: **a** Lovejoy et al.^29^, **b** Tague and Lovejoy^18^, **c** Häusler and Schmid^19^, **d** Brassey et al.^20^; Sts 14: **e** Häusler and Schmid^19^, **f** Berge and Goularas^17^; **g** modern human female pelvis and fetal head (in inset); MH2: **h** Kibii et al.^21^, **i** Laudicina et al.^22^. All pelves are seen in a view perpendicular to the pelvic inlet. Scale bar 5 cm.

Our bony FEA simulations showed a similar outcome for all three australopithecines and all pelvic reconstructions, including A.L. 288-1 (*Australopithecus afarensis*, dated to 3.18 Ma), Sts 14 (*A. africanus*, 2.6-2.1 Ma), and MH2 (*A. sediba*, 1.98 Ma) (Fig. 3). This suggests that our results are robust despite the potential biases associated with fossil reconstructions. Overall pelvic shape, therefore, seems to be of minor importance compared with the actual capacity of the birth canal relative to fetal head size. In fact, all three female australopithecine pelves are of small body size and have a comparably large birth canal cross-sectional area (Table 1).

**Table 1:** Obstetrical analysis of the finite element simulations of the fetopelvic dyads.

Pelvic reconstruction	Pelvic inlet area [cm^2^]	Pelvic inlet AP [mm]	Pelvic inlet TV [mm]	Neonatal brain size [grams]	Delivery outcome of bony simulations	Level of arrest	Nutation [mm]^a^	Cephalo-pelvic gap at inlet [mm]	Delivery outcome taking soft tissue into account
A.L. 288-1, Lovejoy et al.^29^	79	72.4	128	110	Eutocic	–	20	3.9	Dystocic
				145	Dystocic	Inlet	–	0.9	Dystocic
				180	Dystocic	Inlet	–	0.0	Dystocic
A.L. 288-1,Tague and Lovejoy^18^	83	76	132	110	Eutocic	–	20	5.5	Dystocic
				145	Eutocic	–	20	3.0	Dystocic
				180	Dystocic	Inlet	–	0.2	Dystocic
A.L. 288-1, Haeusler and Schmid^19^	86	81	123	110	Eutocic	–	12	7.5	Eutocic
				145	Eutocic	–	12	4.5	Dystocic
				180	Dystocic	Inlet	–	2.1	Dystocic
A.L. 288-1, Brassey et al.^20^	79	80	128.5	110	Eutocic	–	0	7.0	Eutocic
				145	Eutocic	–	6	4.3	Dystocic
				180	Dystocic	Inlet	–	0.5	Dystocic
Sts 14, Haeusler and Schmid^19^	72	89	101	110	Eutocic	–	11	7.6	Eutocic
				145	Eutocic	–	11	5.9	Dystocic
				180	Dystocic	Midplane	–	3.4	Dystocic
Sts 14, Berge and Goularas^17^	77	83	116.8	110	Eutocic	–	20	8.6	Eutocic
				145	Eutocic	–	20	6.2	Dystocic
				180	Dystocic	Midplane	–	2.7	Dystocic
MH2, Kibii et al.^21^ and Laudicina et al.^22^	87	81.7	117.6	110	Eutocic	–	8	8.0	Eutocic
				145	Eutocic	–	13	5.1	Dystocic
				180	Dystocic	Inlet	–	2.0	Dystocic
Modern human female		116.1	136.1	368	Eutocic	–	4	11.3	Eutocic

^a^Nutation represents outlet expansion by backward rotation of the caudal tip of the sacrum.

**Fig. 4:**
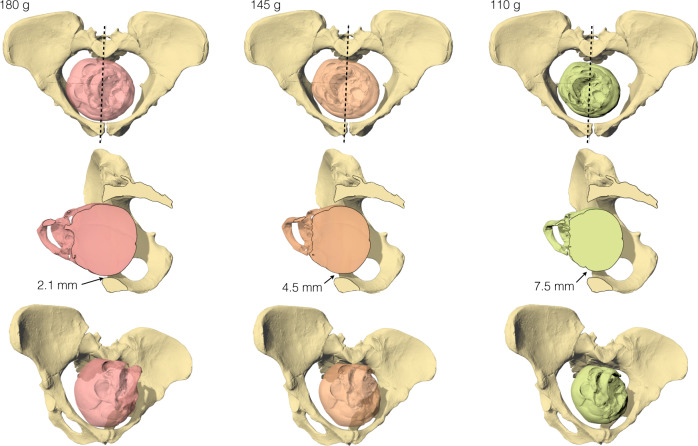
Engagement of the fetal head in the pelvic inlet of A.L. 288-1, pelvic reconstruction of Häusler and Schmid^19^. The in silico simulation shows that only the 110 g fetal head size leaves sufficient space (i.e., >7–10.6 mm) for fetopelvic soft tissue. The best cephalopelvic fit is obtained with a slightly oblique head presentation at the pelvic inlet, and the maximum constriction occurs in a para-sagittal plane (dashed line). Top row: view perpendicular to pelvic inlet. Middle row: right lateral view, clipped at the plane of maximum constriction; the figures indicate the width of the gap between the fetal skull and the maternal pelvis. Bottom row: oblique perspective view.

All bony FEA simulations using a 180 g fetal brain size resulted in dystocic birth. The descent stopped at either the inlet or midplane even when the biparietal diameter of the fetal skull was slightly smaller than the anteroposterior diameter of the pelvis due to the eccentric alignment of the fetal head with the heart-shaped pelvic canal (Fig. 4, Supplementary Figs. 1–8, Supplementary Tables 2 and 3). A mid-arrest was observed in our bony birth simulations of the mother-infant dyads of the two Sts 14 pelvic reconstructions with a 180 g brain size (Table 1, Supplementary Fig. 1). This suggests that mean neonatal brain size of *Australopithecus* was smaller than predicted by a neonatal-to-adult brain size ratio for a general primate. Conversely, all australopithecine pelves allowed the passage of a 145 g fetal brain size except for Lovejoy’s^29^ reconstruction of A.L. 288-1, which possessed the smallest anteroposterior diameter. The pelvic constriction was slightly reduced after scaling the model to the dimensions published by Tague and Lovejoy^18^ (see Methods). All bony simulations with a 110 g fetal head size resulted in an eutocic birth.

### Fetopelvic soft tissue thickness

Since most cephalopelvic constraints of our bony simulations occurred at the pelvic inlet, we proceeded to analyse the width of the space between the bony pelvis and the fetal skull. Our in silico simulations confirmed previous studies^18,22^ that were based on chimpanzee-sized fetal heads (corresponding to a ca. 155 g = 162 cm^3^ brain size) by finding no direct bony obstruction in A.L. 288-1 (*A. afarensis*) or MH2 (*A. sediba*). However, we observed a minimum gap of only 0.7–4.1 mm between the external bony surface of a 155 g fetal skull and the maternal pelvic inlet of these reconstructions. With the 145 g fetal head that we used in our FEA simulations, this cephalopelvic gap increased to 0.9–4.5 mm in A.L. 288-1, to 5.9–6.2 mm in Sts 14, and to 3.2–5.1 mm in MH2, while with a 110 g fetal head, the gap became 3.9–7.5 mm in A.L. 288-1, 7.6–8.6 mm in Sts 14, and 8.0 mm in MH2 (Table 1). This contrasts to the average fetopelvic soft tissue thickness of about 9.5 mm (associated with emergency Caesarean section in modern humans) to 12.6 mm (associated with vaginal delivery) to which it is compressed in the birth canal^30^ (see Methods); the fetopelvic soft tissue consists of the fetal scalp, the amnion, chorion and the mean of the retropubic and presacral soft tissue of the mother’s pelvic canal. Consistent with this, we observed a mean fetopelvic soft tissue thickness in the midsagittal plane of 11.3 mm in our in silico birth simulation of an average modern human female pelvis paired with an average sized fetal skull (Supplementary Fig. 9). Adjusting for the smaller body size of australopithecines, this suggests on average a fetopelvic soft tissue thickness of about 7.0–10.6 mm at the narrowest places in the birth canal of these early hominins (see Methods).

Even if the minimum fetopelvic soft tissue thickness based on values observed with Caesarean sections in modern humans is used as a conservative estimate, in combination with the size and shape of the birth canal, the average neonatal brain size of *Australopithecus* seems to have been closer to 110 g rather than 145 g. Given a presumed variation around this mean, some individuals might of course have been able to give birth to larger infants. In fact, birth outcome depends on a variety of multifaceted factors in addition to fetus size and soft tissue characteristics, including type of presentation, parity, uterine activity, ligamentous relaxation, birth posture, asynclitism, etc. However, to guarantee survival of the species as a whole, the average values need to be considered rather than what is feasible under exceptional circumstances in particular individuals.

### Influence of fetal head moulding

In humans, the fetal head often undergoes significant deformation caused by cranial moulding during birth. This typically leads to a reduction of the vertical diameter and a concomitant elongation of the occipitofrontal diameter, while biparietal breadth remains nearly unaffected^31,32^. Conversely, substantial moulding has not been reported for non-human primates, which is likely due to the more advanced closure of the fontanels and cranial sutures at birth^26,33^. In addition, high-resolution CT scans of the Taung skull^34^ did not confirm earlier claims based on medical CT images^35^ suggesting delayed fusion of cranial sutures in australopithecines. In our simulations with a dystocic outcome, the greatest compressive stresses were observed laterally at the parietal bones of the australopithecine fetal skulls. This is, however, a dimension that is barely affected by moulding since the rigidity of the basicranium prevents substantial deformation of fetal head breadth in primates. Thus, even if fetal head moulding would have already been present in *Australopithecus*, the passage of a fetus with an average brain size of 145 g or larger would have been difficult, if not impossible.

### Ligamentous laxity

In smaller quadrupedal primates with relatively large fetal heads compared with the maternal pelvic dimensions such as Old World monkeys like *Papio* and New World monkeys like *Saimiri* (and other small mammals), the symphysis can open to increase pelvic inlet area up to 30% and 100%, respectively^26^. In contrast, ligamentous laxity at the symphysis seems to be negligible in great apes and humans given that the two hipbones often fuse in chimpanzees at the symphysis in both sexes^27^, whilst in humans the symphysis widens on average by only 3 mm towards the end of pregnancy, rendering the inlet as a virtually undeformable bony ring^26,28^. This rigidity seems to be related to the greater body size and bipedal locomotion of great apes and modern humans, respectively, which increases shearing stress at the pubic symphysis. Furthermore, MRI studies have demonstrated that although the utilization of non-supine birth positions such as kneeling and squatting increases the pelvic diameters of the midplane and outlet, it reduces those of the pelvic inlet^36,37^. In clinical practice, this makes the inlet particularly prone to arrest of labour due to fetopelvic incongruence despite sufficient uterine contractions. Without assistance, this would provoke obstetric disorders ranging from urogenital fistulas to uterine rupture^1^. Fortunately, in contrast to the inlet, both anteroposterior and transverse diameters of the pelvic midplane and outlet can be significantly increased thanks to sacro-iliac joint mobility, particularly in non-supine birth positions^1,36,37^. Birth might therefore still be possible—albeit difficult—in the event of a mid-arrest, as we observed it in our bony FEA birth simulations in Sts 14, where the 180 g fetal heads were entrapped between the ischial spines (Table 1).

### Fetal head rotation

All bony FEA simulations with an eutocic outcome showed a rotation of the fetal head during the descent if they started from a left occiput anterior (LOA) head orientation, which is the typical fetal head presentation at the pelvic inlet in modern humans. The only exception was the dyad of a 110 g fetal head size and the reconstruction of A.L. 288-1 by Brassey et al.^20^. In that particular dyad, the fetal skull did not get into contact with the pelvis due to the relatively straight pelvic canal, and rotation was consequently not induced.

The majority of our australopithecine reconstructions showed a transverse fetal head orientation at the pelvic outlet (Table 1). Such transverse positions are unusual in primates and, more generally, in mammals. The transverse head orientation at the outlet appears to be an artifact of the absence of soft tissue in our models. In fact, not only modern human females with an average-shaped pelvis, but also those with a transversely oval (platypelloid) bony pelvic midplane and outlet (measured before the tip of the sacrum is nutated backward) regularly show an internal rotation into an occiput anterior orientation of the fetal head^38–40^. Nevertheless, our bony birth simulation conducted for a modern human female with average pelvic and fetal head dimensions showed an anomalous 45° rotation of the fetal head into a transverse orientation at the pelvic outlet. Our results thus confirm what has variously been described by obstetricians^38–41^, i.e. that fetal head rotations in the pelvic cavity cannot reliably be simulated based on bony morphology alone.

A crucial role in triggering internal rotation is thought to be played by the pelvic floor muscles. Thus, the slopes of the funnel-shaped inner section of the pelvic floor, which is mainly formed by the three muscles of the levator ani that taper into the sagittally elongated levator hiatus at its bottom, force the fetal head into a sagittal orientation when entering the pelvic cavity, while the lever arm of the fetal skull with respect to the occipitally positioned foramen magnum leads to a simultaneous flexion of the neck^1,38–40^. The rotational moment of the levator ani muscle is reinforced by the backward nutation of the sacrum that stretches the pelvic floor antero-posteriorly (Supplementary Fig. 10). Conversely, if the pelvic floor muscles are flaccid and the fetal head is small and roundish, internal rotation might fail to occur^1,38–40^. During the following phase of expulsion from the birth canal, when the 4 cm deep funnel of the levator ani and the other pelvic floor muscles are rolled out into a 15 cm long soft tissue canal, a persisting transverse head position is usually linked to arrest of labour, excessive stretching of the perineum and severe perineal tears as well as shoulder dystocia in modern humans^1,38–41^. Since early hominins presumably had the same configuration of the pelvic floor as modern humans and great apes, they are expected to show a comparable sagittal head orientation during expulsion from the birth canal.

In conjunction with the outcome of our bony FEA simulation of a modern human dyad with mean fetopelvic dimensions, we therefore conclude that internal rotation is corollary to the evolutionary change from the longitudinally oval pelvic inlet of non-human primates to the heart-shaped form of hominins and that the shape of the lower pelvic planes does not contribute to rotational birth. Consequently, this also challenges the outcome of previous studies that suggested a transverse position of the fetal head at the pelvic outlet in *Australopithecus*^18,22^. More specifically, the markedly different dimensions of the lower pelvis of the four available reconstructions of A.L. 288-1 would not affect fetal head orientation at the outlet, and the same applies to the uncertainties in pelvic outlet shape of Sts 14 and MH2. Likewise, the nearly circular cross-section of the flexed (but also of the fully extended), longitudinally oval fetal head means that the exact shape of the skull is of minor importance. The choice to utilize a fetal head model based on a newborn modern human or a chimpanzee is therefore irrelevant in such simulations (Supplementary Fig. 11), and FEA models that incorporate the maternal pelvic floor musculature and the infant’s body, along with forces simulating uterine contractions, would be required to accurately predict the fetal head orientation at the pelvic outlet.

### Secondary altriciality and the obstetrical dilemma hypothesis

Independent of the evidence for rotational birth, our results imply that australopithecines were (on average) not able to deliver infants with a newborn-to-adult brain size relationship characteristic of great apes. Rather, they likely evolved neonatal-to-adult brain size proportions similar to those of modern humans, thus requiring comparable adaptations like secondary altriciality to diminish the risk of cephalopelvic disproportion. This is based on the outcome of our bony finite-element simulations and the three-dimensional fit of the pelvic inlet only in combination with a range of different fetal head sizes and a conservative estimate for soft tissue thickness. In contrast, the bony dimensions of the lower pelvic planes are secondary with respect to cephalopelvic fit. Because the circumference of the pelvic inlet was preserved in all three fossils, the differences between the various pelvic reconstructions were negligible as variation between the reconstructions in the sagittal diameter resulted in a compensatory widening or narrowing of the transverse diameter. Potential issues in the lower pelvic planes with too small reconstructed pelvic diameters, e.g., due to the incomplete preservation of the sacrum in Sts 14 and uncertainties in ischium shape of MH2, were counterweighted by the ligamentous laxity of the sacroiliac joint and nutation of the sacrum.

Additional support for secondary altriciality, i.e., for neurologically more immature newborns in australopithecines compared with those of non-human primates, comes from the almost human-like, slow brain growth pattern reported for the ~2.4-year-old DIK 1/1 (*A. afarensis*) child^23^. This protracted brain growth has originally been interpreted within the framework of the metabolic hypothesis of human altriciality^23^. In contrast to the obstetrical dilemma hypothesis, this so-called Energetics of Gestation and Growth (EGG) hypothesis posits that the relatively small brain size at birth in humans is the result of a limitation of the energy the mother can invest in fetal growth during pregnancy^9^ rather than the result of biomechanical pelvic constraints relative to fetal head size during birth. This is based on the assumption that fatty acids required for fetal brain growth do not cross the placenta efficiently (but see ref. ^6^). However, brain size in australopithecines was only marginally larger relative to body mass than that inferred for our common ancestor with chimpanzees^15^ and there is consequently no reason to assume that the placenta in australopithecines was less efficient than that of chimpanzees. It is therefore difficult to imagine that metabolic reasons alone would have prevented these early hominins from giving birth to infants with brain sizes of 166–184 g as predicted from newborn-to-adult brain size proportions of a general primate model. Hence, our findings of the presence of pelvic constraints on fetal head size even in the relatively small-brained australopithecines support the original obstetrical dilemma hypothesis^8^ that secondary altriciality in hominins is primarily related to the anteroposterior shortening of the birth canal as an adaptation to bipedalism rather than to metabolic limitations of the placenta, although some combination of these two hypotheses is also conceivable^6^.

Whereas the birth trajectory of chimpanzees and non-human primates in general is nearly straight, a recent 3D simulation of the birth mechanism in chimpanzees^42,43^ challenged the traditional view of a spacious birth canal in great apes^33^ by revealing a similarly tight cephalopelvic fit as in modern humans. The gap between the anterolateral wall of the bony pelvis and the parietal bones of the fetus was demonstrated to be on average only about 1–2 mm wider than in humans, while the distance to the sacrum was considerably narrower^42,43^. Assuming the same cephalopelvic proportions in the last common ancestor of hominins and chimpanzees, even a slightly reduced capacity of the birth canal and an increased curvature of the birth trajectory would have substantially intensified the obstetric selection pressures in australopithecines. Evidence for this is also provided by the presumed male australopithecine pelvic remains discovered in the last decades such as KSD-VP 1/1, StW 431, Sts 65, and MH1 that all possess a narrower greater sciatic notch compared with A.L. 288-1, Sts 14, and MH2^21,44,45^. This higher degree of sexual dimorphism in pelvic shape compared with great apes has been argued to be an adaptation to mitigate the obstetric consequences of a convoluted, tight birth canal^2,6,46^.

**Fig. 5:**
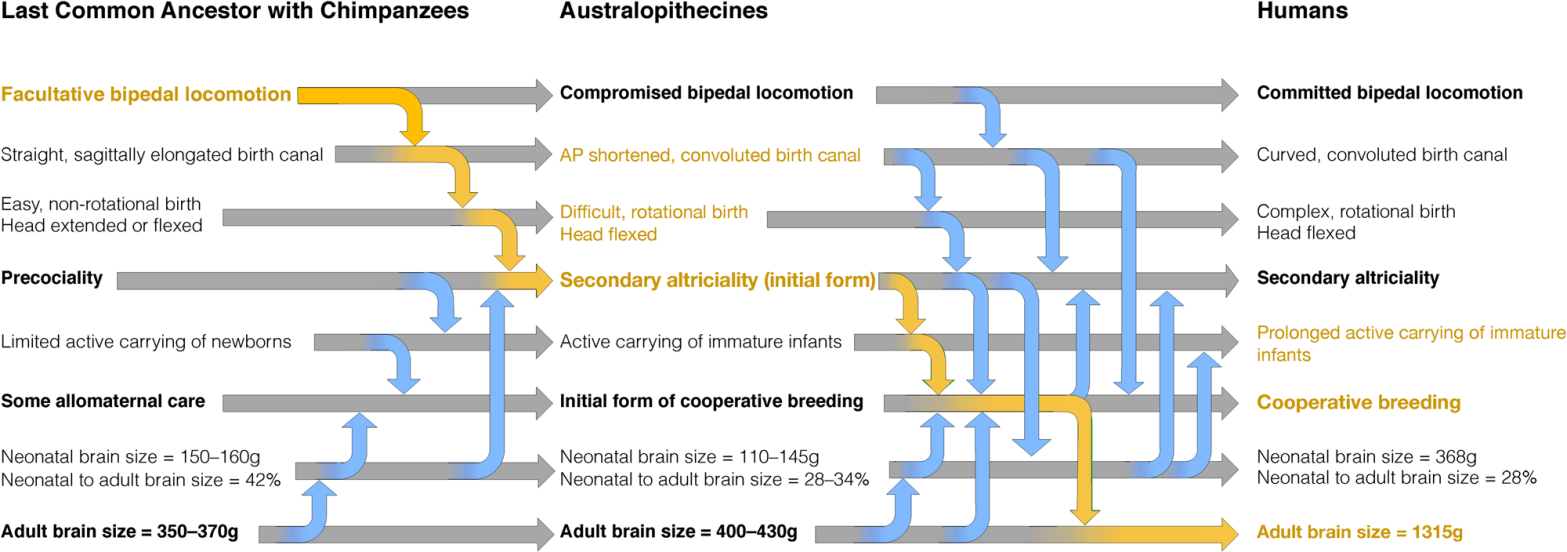
Obstetrically relevant evolutionary changes during hominin evolution and their interrelationship with locomotion and encephalization. Adaptations to bipedal locomotion in early hominins are proposed to be at the origin of an evolutionary cascade (yellow arrows and text) that led to an antero-posteriorly (AP) shortened and convoluted birth canal. As a solution to this increased birth complexity, australopithecines gave birth to secondary altricial newborns with relatively small brain sizes. These neurologically immature newborns had to be carried by family members, thereby necessitating the evolution of cooperative breeding, which represented an exaptation for brain size enlargement in the genus *Homo*. Bold font: main characteristics; gray arrows: evolutionary changes; blue arrows: influencing factors.

Neurologically immature australopithecine infants would have required more assistance, including the need to be actively carried for a prolonged period after birth, suggesting that behaviours like provisioning and cooperative care may have been initiated at this early stage of human evolution before the appearance of the genus *Homo* (Fig. 5). Cooperative breeding has been argued to represent the most plausible exaptation for brain size increase in hominins^47,48^. Such elaborate social behaviours likely established a trend towards prolonged cognitive development that was crucial for the eventual acquisition of human-like intellectual capabilities^49,50^, and it provided an apt environment for the manufacturing of the earliest documented stone tools 3.3 Ma ago at a time long before the appearance of the earliest *Homo* fossils^51^. Consequently, our results suggest that the restructuring of the pelvis due to bipedalism created the selection pressure leading to an initial step towards a human-like life history pattern. As such, both secondary altriciality and rotational birth seem to have ensued from bipedalism rather than from encephalization itself. Hence, it was bipedalism that prepared the adaptive milieu for the drastic encephalization occurring later during the evolution of the genus *Homo*.

## Materials and methods

### Finite-element analysis

The birth simulations were performed with finite-element analyses (FEA) using Radioss 11.0 (www.altair.com). As is typical of FEA modelling, our approach required simplification to understand the effects of various parameters while still capturing the intended physiological phenomenon of birth accurately. Specifically, the complexity of the birth process was reduced to dyad dynamic simulations characterized by a set of solvable differential equations. Deformation of the fetal skull due to collision with the pelvis was enabled by the different assigned material properties and the force of descent applied to the fetal head. A free time step, i.e., a sampling interval Δt between two following cycles, of 0.001 ms was considered for each calculation. The time step was decreased up to 1 × 10^−6^ ms when two surfaces were approaching each other. The decreasing time step permitted the management of the numerical relationship between the pelvis and the fetal head. The resulting animation was recorded at each millisecond. The total computation time of a simulation was between 2 and 4 h depending on the number of processors used (6–12).

### Pelvic meshes

We included all available female *Australopithecus* pelvic reconstructions except for the Sts 14 reconstructions by Robinson^52^ and Abitbol^53^ that were excluded owing to their major anatomical shortcomings^19,45^, while the A.L. 288-1 reconstruction by Schmid^54^ was not available.

For the A.L. 288-1 (*A. afarensis*) pelvis, four different reconstructions were used in the analyses. All of them were based on a mirror-image reconstruction of the missing right hipbone while the crushed sacrum remained uncorrected. The crushed posterior ilium with the sacroiliac joint was only restored in the manual reconstruction of Haeusler and Schmid^19,55^. A corresponding cast was scanned with a high-resolution surface scanner (PT-M4c, Polymetric GmbH, Darmstadt, Germany), and slight discrepancies compared with the published dimensions were corrected by anisotropic scaling of the model for consistency. Lovejoy’s^29^ manual reconstruction of A.L. 288-1 was generously provided by the author as a 3D surface scanner-generated model based on a cast. This reconstruction was said to be the same as that used by Tague and Lovejoy^18^. However, the diameters of the pelvic canal were slightly smaller than the dimensions published by Tague and Lovejoy^18^ (inlet AP 73 vs. 76 mm, ML 128 vs. 132 mm, midplane AP 70 vs. 72 mm, ML 106 vs. 101 mm; AP = anteroposterior diameter, ML = mediolateral diameter) (Table 1 and Supplementary Table 2). We therefore scaled the 3D model of Lovejoy’s^29^ reconstruction by a factor of 1.046 sagittally and 1.033 mediolaterally as well as superoinferiorly to obtain the dimensions of the Tague and Lovejoy^18^ reconstruction. Because there was no explanation for the discrepancy between the two, except for possible anisotropic shrinkage of the casts, both variants were used in our simulations. The virtual reconstruction of the A.L. 288-1 pelvis by Brassey et al.^20^ was available as a 3D model from Figshare (10.6084/m9.figshare.c.3462618).

The Sts 14 (*A. africanus*) pelvis was available in the form of two different reconstructions. The manual reconstruction by Häusler and Schmid^19^ was based on all preserved elements of the pelvis. The ischial tuberosities and the caudal three sacral vertebrae were reconstructed using the proportions of A.L. 288-1 as a reference, while sacral curvature was extrapolated from the curvature of the cranial half of the sacrum. The virtual reconstruction by Berge and Goularas^17^ is a symmetrised mosaic of elements mirrored from the left and right hipbones, while the ischial tuberosities and the caudal sacral vertebrae were not restored.

For MH2 (*A. sediba*), also two reconstructions were available, both of which were based on mirroring the preserved right sacrum fragment and the right ilium and pubis, while the ischium was based on the morphology of MH1. A cast of the reconstruction by Kibii et al.^21^ (provided by Peter Schmid) was scanned with a PT-M4c high-resolution surface scanner. Since this model also showed slight discrepancies of the birth canal dimensions when compared with the published diameters, we scaled it accordingly for consistency. The MH2 reconstructions by Laudicina et al.^22^ and the scaled version of Kibii et al.^21^ showed nearly identical dimensions of the pelvic canal. However, because the sacrum promontorium was not reconstructed by Laudicina et al.^22^, only the version of Kibii et al.^21^ was used in the simulations.

The sacra of Lovejoy’s A.L. 288-1 reconstructions and that of Kibii et al.^21^ were isolated from the hipbones via segmentation conducted in Geomagic (www.3dsystems.com) to render a mobile sacro-iliac joint. All 3D models were re-meshed in Hypermesh 12.0 (www.altair.com) to generate shell elements of an average size of 1 mm and to eliminate mesh inconsistencies, duplicated faces and other artifacts.

### Fetal skull meshes

Our fetal skull model used for the bony FEA simulations was based on a medical CT scan of a human fetus at 35 weeks of gestation (local ethics committee number: 1d-RCB 2011-A00072-39). The CT scan was performed with a 16 slice Siemens SOMATOM Definition Flash strip scanner with 0.6 mm slice thickness. The CT images were segmented in Mimics 12.3 (www.materialise.com). The generated polygonal mesh of the fetal head was re-meshed in Hypermesh 12.0 (www.altair.com) to produce 18,000 shell elements with an average size of 1 mm (Fig. 1). The fetal head model was then scaled to conform to the brain masses of 180, 145, and 110 g using the neurocranial proportions of a chimpanzee neonate^24^ (in fact, fetal neurocranial proportions of chimpanzees, humans, and those predicted for Taung are almost identical)^56^. This yielded fetal heads with biparietal diameters of 75, 70, and 64 mm, respectively, and occipito-frontal diameters of 87, 81, and 75 mm, respectively (Supplementary Table 3).

### Models and data setting

Material properties were assigned according to the fetal head model of Lapeer and Prager^57^, with a Young’s modulus for the stiffness of the skull bones of E = 3,800 MPa and E = 200 MPa for the fontanels, which corresponds to material properties close to that of cartilage^58,59^. The skull and the fontanels were considered as linear elastic materials^57^. The power of descent of the fetus was modeled by applying the force of gravity to the centre of the fetal head^60^ with the vector of the force of gravity being oriented perpendicular to the pelvic inlet. This relatively weak expulsion force prevented excessive deformation of the fetal skull but was sufficient to initiate the model and guide the fetal head through the pelvis without prescribing any particular trajectory for the descent. Our simulations were intended to ultimately reflect the basic mechanical behaviour of the fetal head of the common ancestor of chimpanzees and humans as well as *Australopithecus*, which is presumed to show only minimal moulding during labour similar to that of chimpanzees^33^.

Material properties of cortical bone equal to E = 18,000 MPa were assigned to the hipbones and to the sacrum^61^. The Poisson’s ratio ν was 0.3 for all parts and the density 2.1 g/cm^3^. The hipbones and the sacrum were considered as rigid bodies employing the Johnson–Cook model, a model used to represent the non-linear behaviour of bone during dynamic simulations^56,62^. The modulus of rigidity G was determined according to Hooke’s law as G = E/2(1 + ν). The hipbones were fixed in all 6 degrees of freedom. The contact boundary was set to 1 mm.

During delivery, the modern human sacrum can nutate (i.e., rotate) thanks to the hormonally mediated laxity of the ligaments of the sacro-iliac joint causing a posterior displacement of its apex by up to 2–2.5 cm^39,63,64^ (Supplementary Fig. 12). We therefore modeled the sacro-iliac junction by a spring located in the centre of the sacro-iliac joint (Fig. 1) and allowed movements only around the transverse axis with 1 degree of freedom^65^. The nutation of the sacrum was associated with an increasing resistance up to a maximum mobility of 2 cm. Thus, until 10° of rotation, the movement was free, while between 10° and 13.5° the stiffness of the rotation increased from 0.1 Nm^−1^ to 1000 Nm^−1^, corresponding to a complete limitation of the mobility of the sacrum.

We started our simulations with the fetal head in a left occiput anterior (LOA) orientation, in which the fetal head is flexed and the occiput points to the left pubis of the maternal pelvis^1^. This is the most common fetal head presentation at the pelvic inlet in modern humans, and ~50% of chimpanzee births occur with a flexed head^66,67^. A mentum anterior (i.e., a face) presentation as it is characteristic of non-human primates^26^ is deemed less likely because of the greater similarity of the australopithecine pelvic shape to that of humans rather than to that of non-human primates. Furthermore, face presentations are usually associated with dystocic birth in modern humans^1^. The LOA presentation is also the most plausible orientation according to Gauss’ principle of least constraint^68^, which has been applied to obstetrics by Joulin^69^ and Sellheim^70^. This principle predicts alignment of the longitudinal axis of the fetal head with the widest pelvic diameter. The predominance of the LOA presentation compared with a right occiput anterior position (ROA) has been associated with the location of the rectum on the left side of the pelvic canal^71^. Because australopithecines already possessed a well-developed lumbar lordosis that projected into the abdominal cavity^72^ and the pelvic inlet was heart-shaped as in modern humans due to the protruding sacral promontorium, this also predicts a LOA head position at the pelvic inlet of these early hominins^19^.

To test our approach, we used a birth simulation for an average modern human female pelvis and a standard neonate skull. To avoid effects of relaxed selection due to the introduction of Caesarean sections^73^, age-related changes in pelvic dimensions^74^ and macrosomic offspring due to secular trends in obesity^75^ we used fetopelvic dimensions typical for the 19th century of Central Europe. The mean pelvic inlet of 15 reproductive-aged females of the Weisbach collection (Natural History Museum Vienna) had a sagittal diameter of 116.1 mm and a transverse diameter of 136.1 mm. This collection was assembled by Augustin Weisbach (1837–1914) in the late 19th century from military personnel of the Austro-Hungarian army and thus consists of individuals of known age and sex that were fit to serve and had no pathologies affecting skeletal growth and development^76^. The model for the neonate skull was obtained from 3D surface scans of a modern human neonate (A.H. Schultz collection, Anthropological Institute, University of Zürich) which has been scaled to match the mean neonatal brain mass of 368 g (389 cm^3^; N = 79)^77,78^ with brain size-to-skull proportions of a 2-day-old CT-scanned neonate^79^ (Supplementary Table 3).

### Estimation of soft tissue contribution to cephalopelvic fit

During labour, soft tissue in the birth canal can be compressed by the fetal head only to a certain degree. Using intrapartum transperineal ultrasound, retropubic tissue thickness in humans during vaginal delivery (measured as the shortest distance between the outer capsule of the pubic symphysis and the outer surface of the skin of the fetal head) has been determined as 11.6 ± 3.2 mm (*N* = 59), while Caesarean section was associated with a retropubic tissue thickness of 9.4 ± 2.5 mm (*N* = 23)^30^. To this, we added the thickness of the skin of the fetal head as 1.8 mm, measured on intrapartum MRI scans^31,80,81^. Maternal soft tissue thickness in front of the sacrum is slightly thinner with 6.0–10 mm measured on sagittal plane intrapartum MRI scans^31,80–82^, which is supported by intrapartum X-rays^32,83^. If the fetal head is centred within the birth canal, this implies a mean fetopelvic soft tissue thickness of about 10.6–12.6 mm for vaginal delivery and a lower limit of 9.5 mm (associated with Caesarean section). Given that the body mass of female australopithecines is about 40%–60% of modern human females and that linear dimensions scale to the cube root of body volume and thus body mass, the average fetopelvic tissue thickness of australopithecines can be approximated to between $\root 3 \of {0.4}$ × 9.5 mm and $\root 3 \of {0.6}$ × 12.6 mm = 7.0 mm to 10.6 mm. The analyses of the cephalopelvic fit and fetopelvic tissue thickness was then performed as “in silico simulations” in Rhinoceros 7.0 (www.rhino3d.com) based on 3D surface scans of a modern human and a chimpanzee neonate (A.H. Schultz collection, Anthropological Institute, University of Zürich) scaled to the diameters corresponding to brain masses of 180, 145, 110, and 155 g (corresponding to the average chimpanzee neonatal brain size^24^, only considered in our in silico simulations) (Supplementary Tables 1 and 3)^84,85^.

### Reporting summary

Further information on research design is available in the Nature Research Reporting Summary linked to this article.

## Supplementary information

Supplementary information

Reporting Summary

## Data Availability

All data are available in the main text or the supplementary materials.
