# Author Correction: Dynamic finite-element simulations reveal early origin of complex human birth pattern

**DOI:** 10.1038/s42003-022-03436-3

**Published:** 2022-05-12

**Authors:** Pierre Frémondière, Lionel Thollon, François Marchal, Cinzia Fornai, Nicole M. Webb, Martin Haeusler

**Affiliations:** 1 UMR 7268 ADES, Aix Marseille University, EFS, CNRS, 51 boulevard Pierre Dramard, 13344 Marseille cedex 15, France; 2 Aix Marseille University, School of Midwifery, Faculty of Medical and Paramedical Sciences, 51 boulevard Pierre Dramard, 13344 Marseille cedex 15, France; 3 Aix Marseille University, UMR-T24, 51 boulevard Pierre Dramard, 13344 Marseille cedex 15, France; 4 Institute of Evolutionary Medicine, University of Zürich, Winterthurerstrasse 190, 8057 Zürich, Switzerland; 5 Department of Evolutionary Anthropology, University of Vienna, Djerassiplatz 1, 1030 Wien, Austria; 6 Human Evolution and Archaeological Sciences (HEAS), University of Vienna, Djerassiplatz 1, 1030 Wien, Austria; 7 Senckenberg Research Institute and Natural History Museum Frankfurt, Senckenberganlage 25, 60325 Frankfurt am Main, Germany; 8 Senckenberg Centre for Human Evolution and Palaeoenvironment, Institute of Archaeological Sciences, Eberhard Karls University of Tübingen, Rümelinstrasse 23, 72070 Tübingen, Germany; 9 Present Address: Vienna School of Interdisciplinary Dentistry—VieSID, Wasserzeile 35, 3400 Klosterneuburg, Austria

**Keywords:** Anthropology, Biological anthropology

Correction to: *Communications Biology* 10.1038/s42003-022-03321-z, published online 19 April 2022.

The original versions of this article and the Supplementary Information were missing the affiliation “Human Evolution and Archaeological Sciences (HEAS), University of Vienna, Djerassiplatz 1, 1030 Wien, Austria” for author, Cinzia Fornai:

Pierre Frémondière^1,2,9^, Lionel Thollon^3^, François Marchal^1^, Cinzia Fornai^4,5,8^, Nicole M. Webb^4,6,7,9^ & Martin Haeusler^4,9^

1 UMR 7268 ADES, Aix Marseille University, EFS, CNRS, 51 boulevard Pierre Dramard, 13344 Marseille cedex 15, France.

2 Aix Marseille University, School of Midwifery, Faculty of Medical and Paramedical Sciences, 51 boulevard Pierre Dramard, 13344 Marseille cedex 15, France.

3 Aix Marseille University, UMRT24, 51 boulevard Pierre Dramard, 13344 Marseille cedex 15, France.

4 Institute of Evolutionary Medicine, University of Zürich, Winterthurerstrasse 190, 8057 Zürich, Switzerland.

5 Department of Evolutionary Anthropology, University of Vienna, Djerassiplatz 1, 1030 Wien, Austria.

6 Senckenberg Research Institute and Natural History Museum Frankfurt, Senckenberganlage 25, 60325 Frankfurt am Main, Germany.

7 Senckenberg Centre for Human Evolution and Palaeoenvironment, Institute of Archaeological Sciences, Eberhard Karls University of Tübingen, Rümelinstrasse 23, 72070 Tübingen, Germany.

8 Present address: Vienna School of Interdisciplinary Dentistry—VieSID, Wasserzeile 35, 3400 Klosterneuburg, Austria.

9 These authors contributed equally: Pierre Frémondière, Nicole M. Webb, Martin Haeusler.

This error has been corrected in the HTML and PDF versions of the Article, along with the Supplementary Information, as follows:

Pierre Frémondière^1,2,10^, Lionel Thollon^3^, François Marchal^1^, Cinzia Fornai^4,5,6,9^, Nicole M. Webb^4,7,8,10^ & Martin Haeusler ^4,10^

1 UMR 7268 ADES, Aix Marseille University, EFS, CNRS, 51 boulevard Pierre Dramard, 13344 Marseille cedex 15, France.

2 Aix Marseille University, School of Midwifery, Faculty of Medical and Paramedical Sciences, 51 boulevard Pierre Dramard, 13344 Marseille cedex 15, France.

3 Aix Marseille University, UMRT24, 51 boulevard Pierre Dramard, 13344 Marseille cedex 15, France.

4 Institute of Evolutionary Medicine, University of Zürich, Winterthurerstrasse 190, 8057 Zürich, Switzerland.

5 Department of Evolutionary Anthropology, University of Vienna, Djerassiplatz 1, 1030 Wien, Austria.

6 Human Evolution and Archaeological Sciences (HEAS), University of Vienna, Djerassiplatz 1, 1030 Wien, Austria.

7 Senckenberg Research Institute and Natural History Museum Frankfurt, Senckenberganlage 25, 60325 Frankfurt am Main, Germany.

8 Senckenberg Centre for Human Evolution and Palaeoenvironment, Institute of Archaeological Sciences, Eberhard Karls University of Tübingen, Rümelinstrasse 23, 72070 Tübingen, Germany.

9 Present address: Vienna School of Interdisciplinary Dentistry—VieSID, Wasserzeile 35, 3400 Klosterneuburg, Austria.

10 These authors contributed equally: Pierre Frémondière, Nicole M. Webb, Martin Haeusler.

## Supplementary information

Updated Supplementary Information

