# Supplementary material for: Dynamic finite-element simulations reveal early origin of complex human birth pattern
Source: Commun Biol. 2022 Apr 19;5:377. doi: 10.1038/s42003-022-03321-z (PMC9018746; doi:10.1038/s42003-022-03321-z)
Supplement: Supplementary file 1 — Supplementary information [file 42003_2022_3321_MOESM1_ESM.pdf]

Supplementary Information

Dynamic finite-element simulations reveal early origin of complex human birth pattern

Pierre Frémondière [1,2,10], Lionel Thollon[3], François Marchal[1] , Cinzia Fornai [4,5,6,9], Nicole M. Webb[4,7,8,10] & Martin Haeusler [4,10]

[1] UMR 7268 ADES, Aix Marseille University, EFS, CNRS, 51 boulevard Pierre Dramard, 13344 Marseille cedex 15, France.
[2] Aix Marseille University, School of Midwifery, Faculty of Medical and Paramedical Sciences, 51 boulevard Pierre Dramard, 13344 Marseille cedex 15, France.
[3] Aix Marseille University, UMRT24, 51 boulevard Pierre Dramard, 13344 Marseille cedex 15, France.
[4] Institute of Evolutionary Medicine, University of Zürich, Winterthurerstrasse 190, 8057 Zürich, Switzerland.
[5] Department of Evolutionary Anthropology, University of Vienna, Djerassiplatz 1, 1030 Wien, Austria.
[6] Human Evolution and Archaeological Sciences (HEAS), University of Vienna, Djerassiplatz 1, 1030 Wien, Austria.
[7] Senckenberg Research Institute and Natural History Museum Frankfurt, Senckenberganlage 25, 60325 Frankfurt am Main, Germany.
[8] Senckenberg Centre for Human Evolution and Palaeoenvironment, Institute of Archaeological Sciences, Eberhard Karls University of Tübingen, Rümelinstrasse 23, 72070 Tübingen, Germany.
[9] Present address: Vienna School of Interdisciplinary Dentistry—VieSID, Wasserzeile 35, 3400 Klosterneuburg, Austria.
[10] These authors contributed equally: Pierre Frémondière, Nicole M. Webb, Martin Haeusler

*Correspondence to: pierre.fremondiere@univ-amu.fr; martin.haeusler@iem.uzh.ch

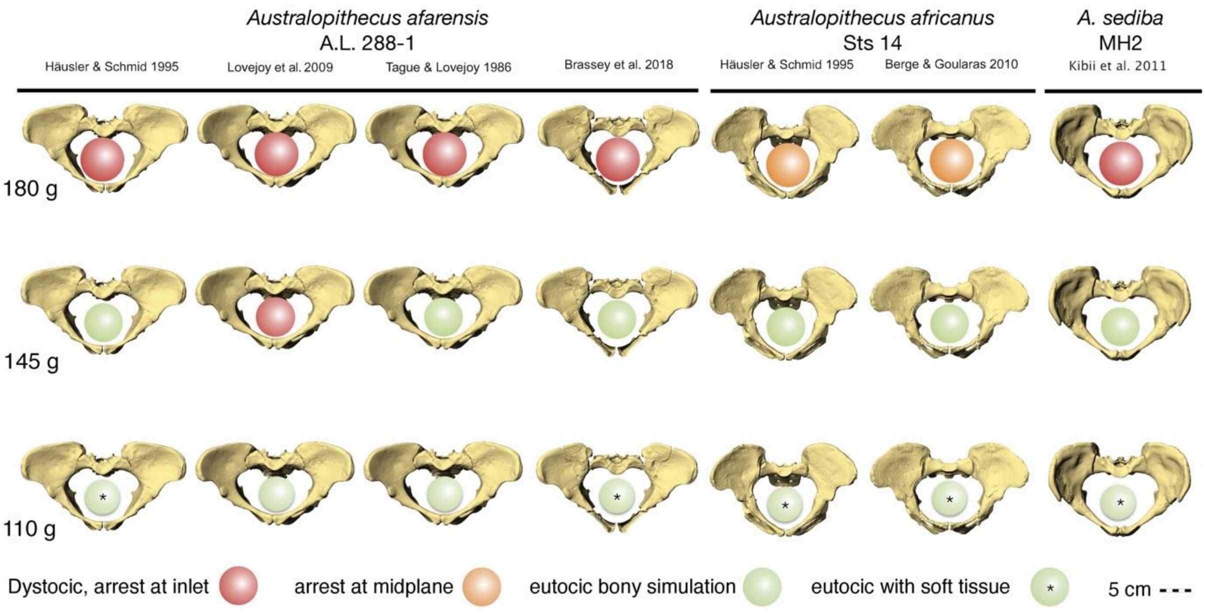

Supplementary Fig. 1. Outcome of bony finite-element (FEA) birth simulations in *Australopithecus*. Simulations based on a 180 g fetal brain size (top), 145 g (middle), 110g (bottom) for the different pelvic reconstructions of A.L. 288-1 (*A. afarensis*)[18-20,29], Sts 14 (*A. africanus*)[17,19], and MH2 (*A. sediba*)[21]. The MH2 reconstruction of Laudicina et al.[22] was not included in the FEA simulations because of the incomplete sacrum; otherwise the birth canal dimensions are, however, virtually identical to the reconstruction of Kibii et al.[21]. A red fetal head signifies a dystocic birth with arrest at the pelvic inlet, orange represents an arrest at the midplane, and green colour shows an eutocic outcome of the bony simulation, while asterisks mark dyads that are also eutocic if fetopelvic soft tissue thickness is taken into account (i.e., minimum thickness > 7–10.6 mm). View perpendicular to the pelvic inlet, scale bar 5 cm.

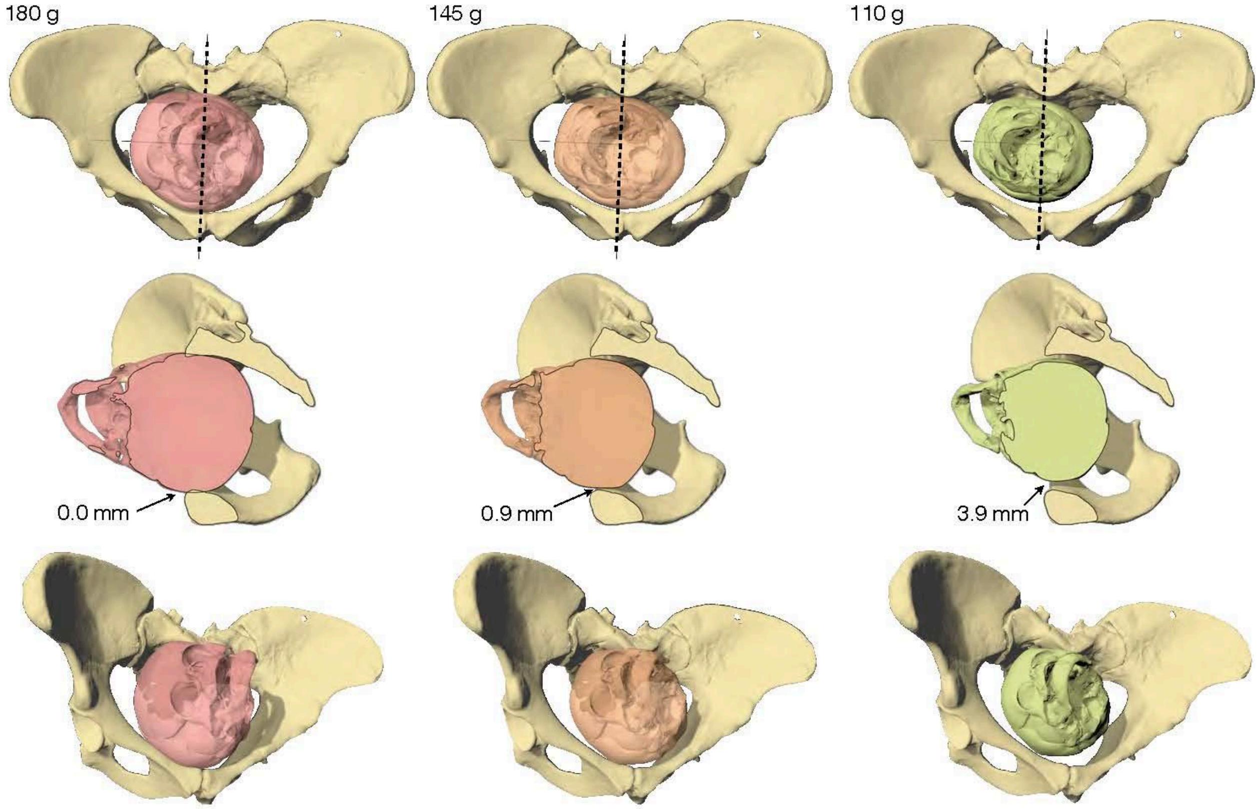

Supplementary Fig. 2. Engagement of the fetal head in the pelvic inlet in A.L. 288-1, pelvic reconstruction of Lovejoy et al.[29]. The *in silico* simulation shows that none of the three different fetal head sizes leaves sufficient space for fetopelvic soft tissue (i.e., > 7.0–10.6 mm). The best cephalopelvic fit is obtained with a slightly oblique head presentation at the pelvic inlet, and the maximum constriction occurs in a para-sagittal plane (dashed line). Top row: view perpendicular to pelvic inlet. Middle row: right lateral view, clipped at the plane of maximum constriction; the figures indicate the width of the gap between the fetal skull and the maternal pelvis. Bottom row: oblique perspective view. The orientation of the fetal head within the pelvis is identical in all three views for each of the three dyads.

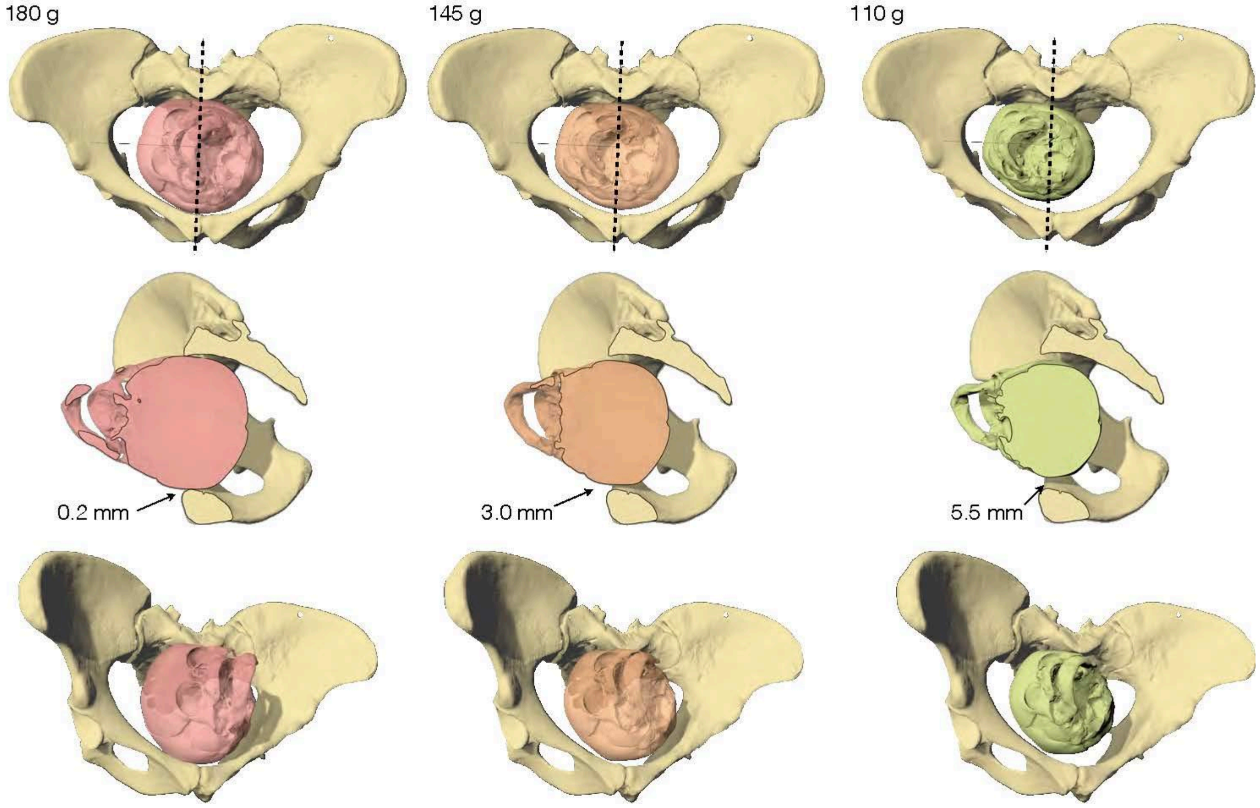

Supplementary Fig. 3. Engagement of the fetal head in the pelvic inlet in A.L. 288-1, Pelvic reconstruction by Tague and Lovejoy[18]. The *in silico* simulation shows that none of the three different fetal head sizes leaves sufficient space (i.e., > 7.0–10.6 mm) for fetopelvic soft tissue. The best cephalopelvic fit is obtained with a slightly oblique head presentation at the pelvic inlet, and the maximum constriction occurs in a para-sagittal plane (dashed line). Top row: view perpendicular to pelvic inlet. Middle row: right lateral view, clipped at the plane of maximum constriction. Bottom row: oblique perspective view. The orientation of the fetal head within the pelvis is identical in all three views for each of the three dyads.

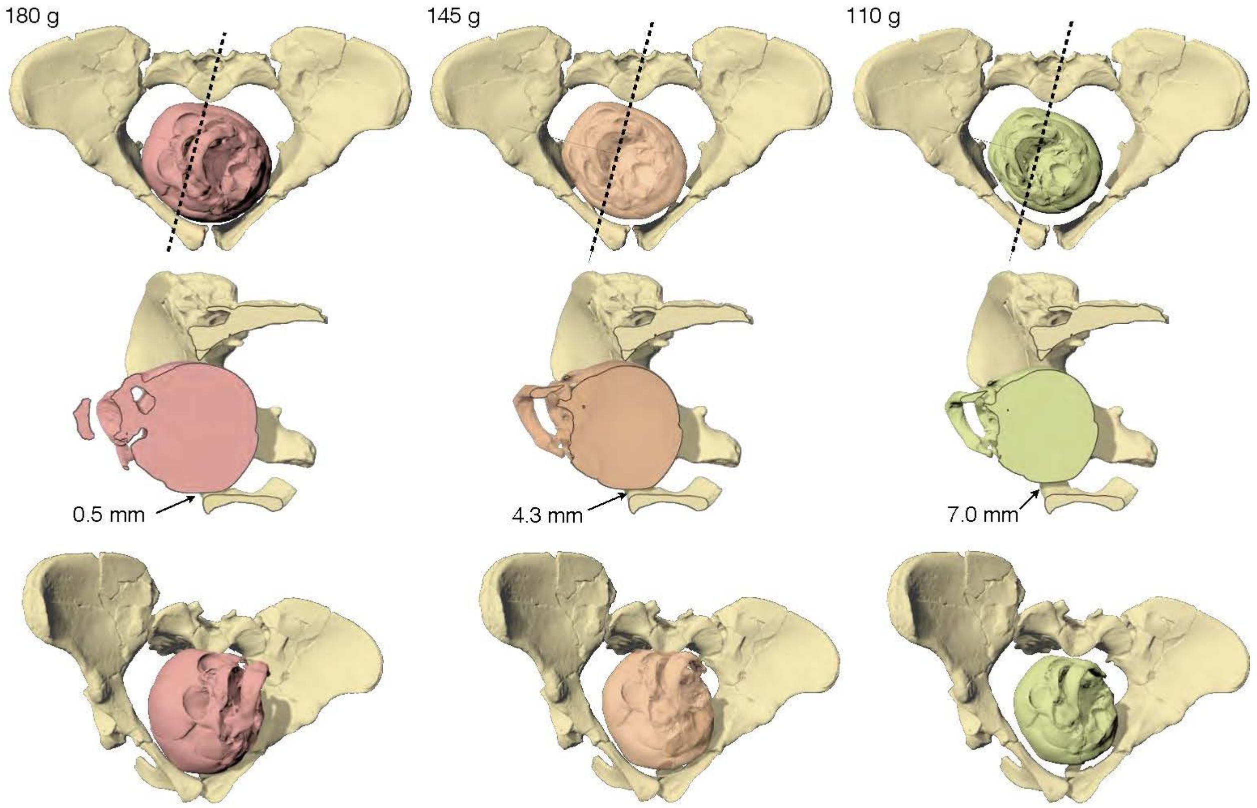

Supplementary Fig. 4. Engagement of the fetal head in the pelvic inlet in A.L. 288-1, pelvic reconstruction of Brassey et al.[20]. The *in silico* simulation shows that only the 110 g fetal head size leaves sufficient space (i.e., > 7.0–10.6 mm) for fetopelvic soft tissue. The best cephalopelvic fit is obtained with a slightly oblique head presentation at the pelvic inlet, and the maximum constriction occurs in a para-sagittal plane (dashed line). Top row: view perpendicular to pelvic inlet. Middle row: right lateral view, clipped at the plane of maximum constriction; the figures indicate the width of the gap between the fetal skull and the maternal pelvis. Bottom row: oblique perspective view. The orientation of the fetal head within the pelvis is identical in all three views for each of the three dyads.

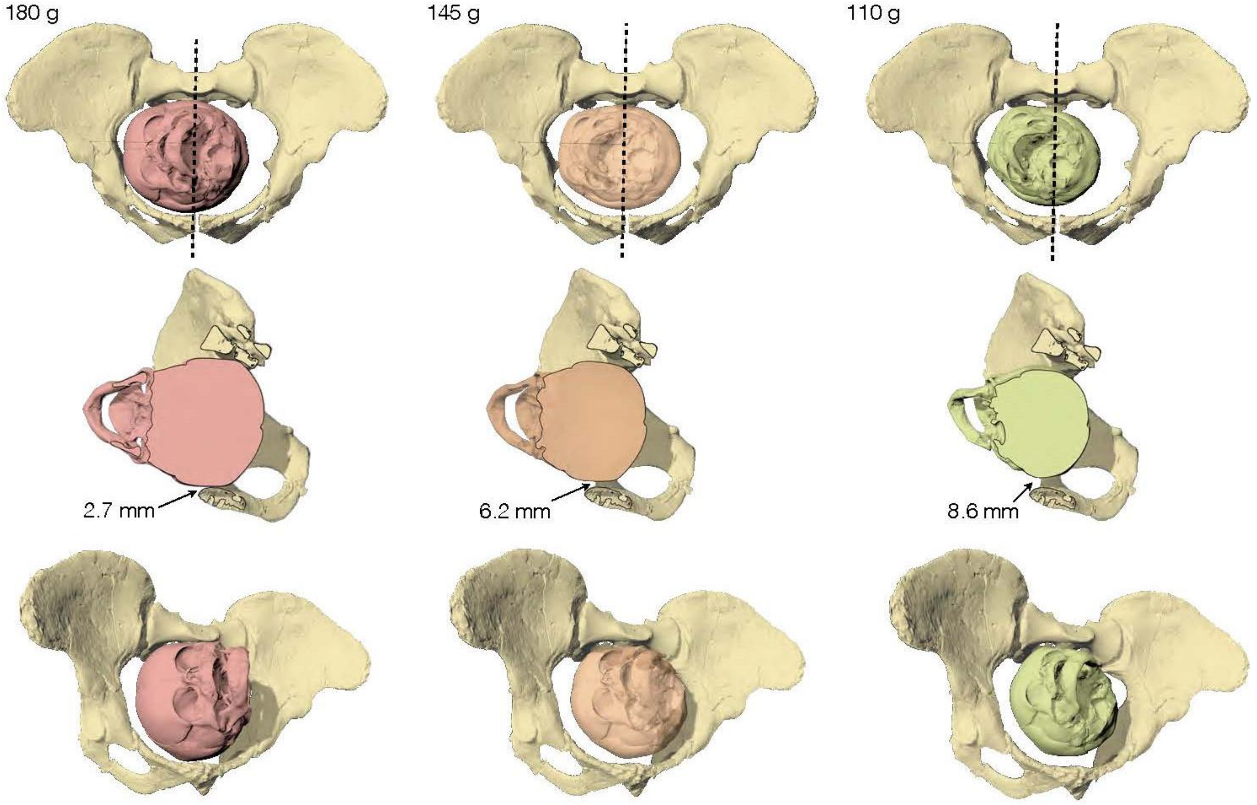

Supplementary Fig. 5. Engagement of the fetal head in the pelvic inlet in Sts 14, pelvic reconstruction of Berge & Goularas[17]. The *in silico* simulation shows that only the 110 g fetal head size leaves sufficient space (i.e., >7.0–10.6 mm) for fetopelvic soft tissue. The best cephalopelvic fit is obtained with a slightly oblique head presentation at the pelvic inlet, and the maximum constriction occurs in a para-sagittal plane (dashed line). Top row: view perpendicular to pelvic inlet. Middle row: right lateral view, clipped at the plane of maximum constriction; the figures indicate the width of the gap between the fetal skull and the maternal pelvis. Bottom row: oblique perspective view. The orientation of the fetal head within the pelvis is identical in all three views for each of the three dyads.

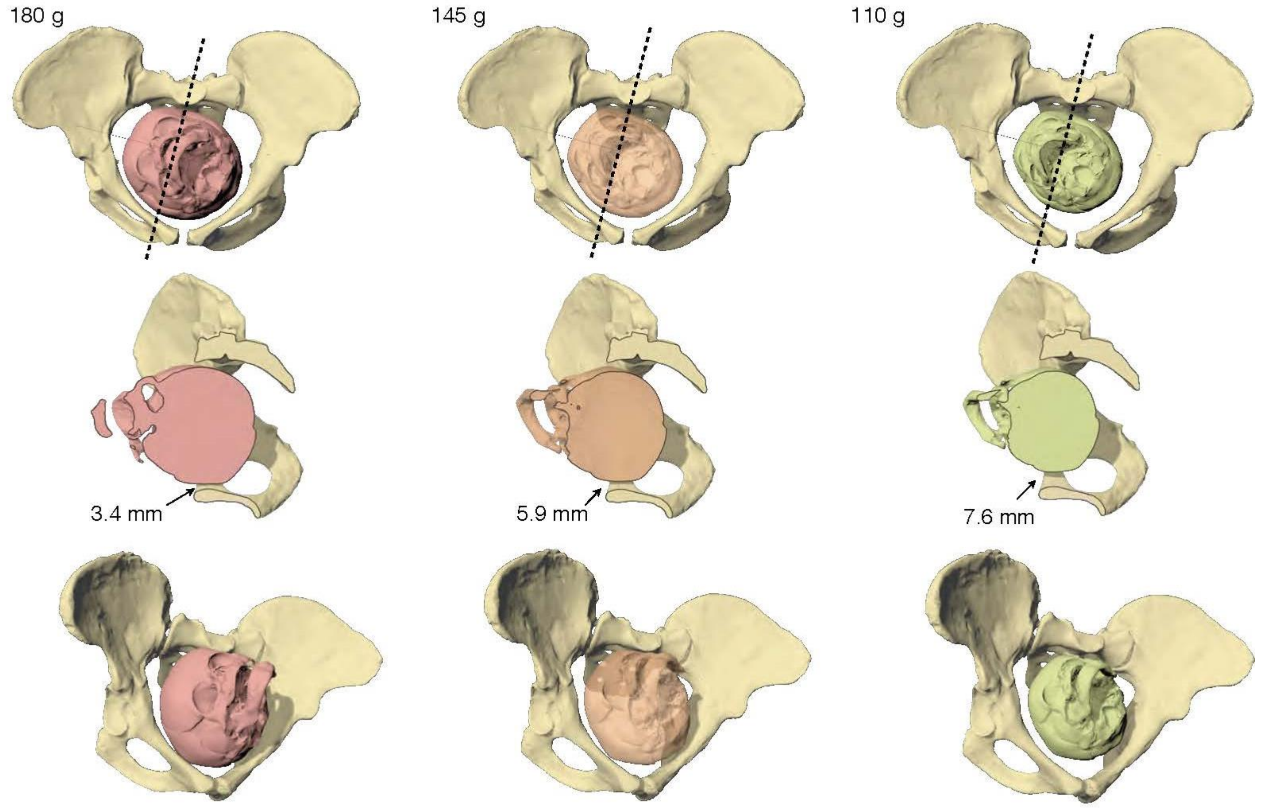

Supplementary Fig. 6. Engagement of the fetal head in the pelvic inlet in Sts 14, pelvic reconstruction of Häusler & Schmid[19]. The *in silico* simulation shows that only the 110 g fetal head size leaves sufficient space (i.e., > 7.0–10.6 mm) for fetopelvic soft tissue. The best cephalopelvic fit is obtained with a slightly oblique head presentation at the pelvic inlet, and the maximum constriction occurs in a para-sagittal plane (dashed line). Top row: view perpendicular to pelvic inlet. Middle row: right lateral view, clipped at the plane of maximum constriction; the figures indicate the width of the gap between the fetal skull and the maternal pelvis. Bottom row: oblique perspective view. The orientation of the fetal head within the pelvis is identical in all three views for each of the three dyads.

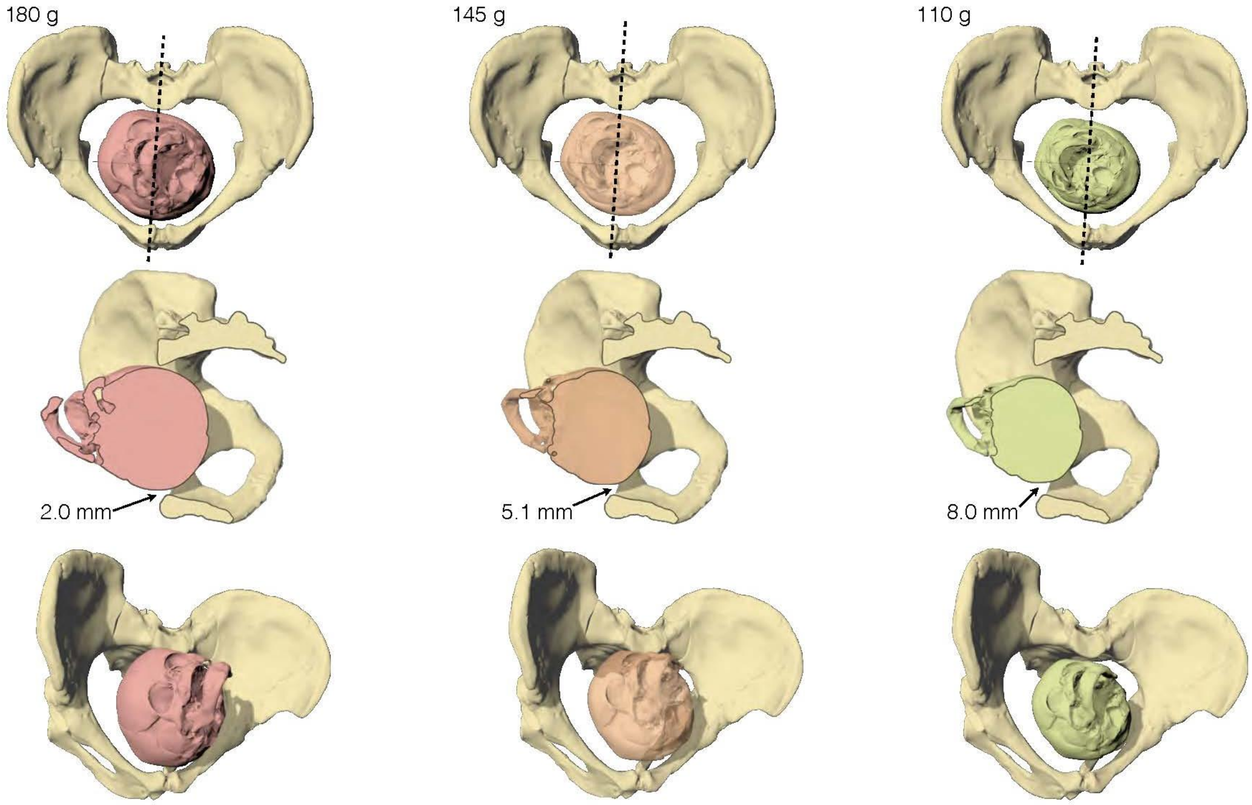

Supplementary Fig. 7. Engagement of the fetal head in the pelvic inlet in MH2, pelvic reconstruction of Kibii et al.[21]. The *in silico* simulation shows that only the 110 g fetal head size leaves sufficient space (i.e., > 7.0–10.6 mm) for fetopelvic soft tissue. The best cephalopelvic fit is obtained with a slightly oblique head presentation at the pelvic inlet, and the maximum constriction occurs in a para-sagittal plane (dashed line). Top row: view perpendicular to pelvic inlet. Middle row: right lateral view, clipped at the plane of maximum constriction; the figures indicate the width of the gap between the fetal skull and the maternal pelvis. Bottom row: oblique perspective view. The orientation of the fetal head within the pelvis is identical in all three views for each of the three dyads.

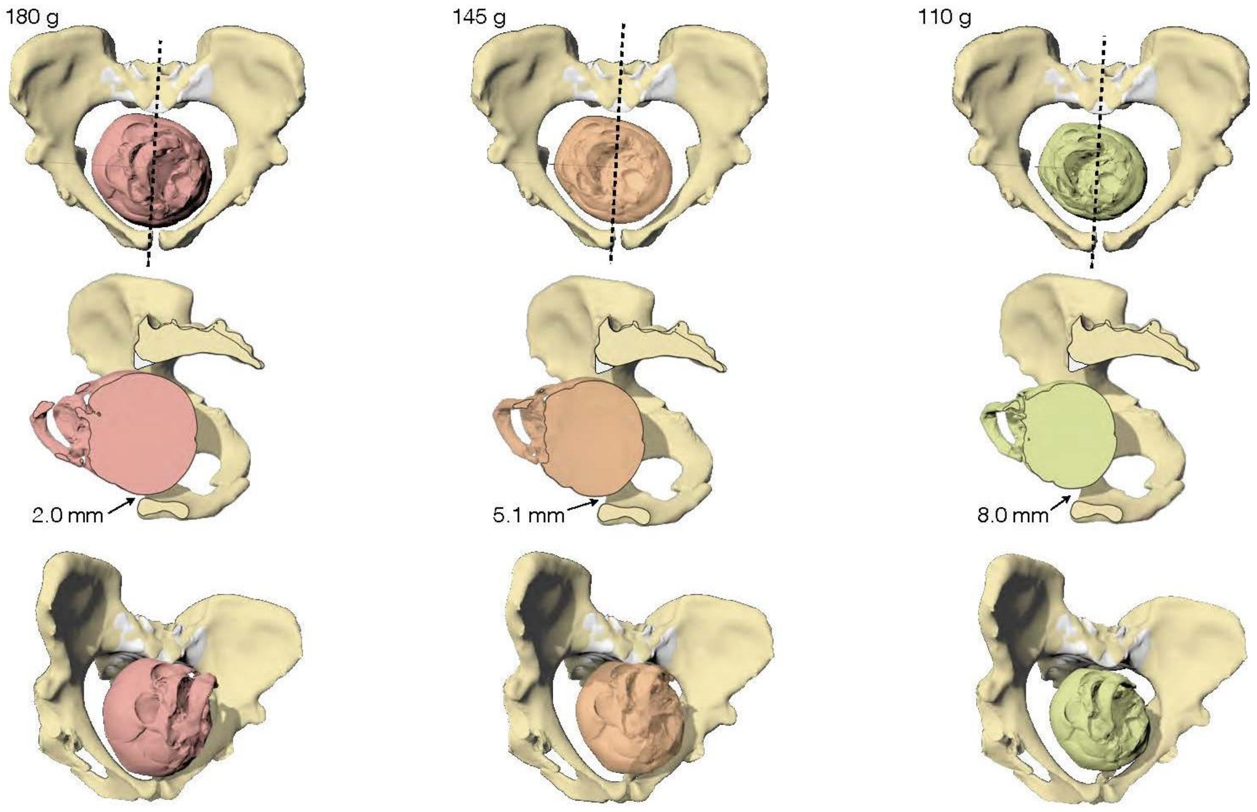

Supplementary Fig. 8. Engagement of the fetal head in the pelvic inlet in MH2, pelvic reconstruction of Laudicina et al.[22]. The sacrum is reconstructed using the sacrum of Kibii et al. [21] (in grey colour). The *in silico* simulation shows that only the 110 g fetal head size leaves sufficient space (i.e., > 7.0–10.6 mm) for fetopelvic soft tissue. The outcome of the in silico simulation is virtually identical to the reconstruction of Kibii et al.[21], and therefore only the latter has been included in the FEA simulations. Top row: frontal view. Middle row: right lateral view, clipped at the plane of maximum constriction; the figures indicate the width of the gap between the fetal skull and the maternal pelvis. Bottom row: oblique perspective view. The orientation of the fetal head within the pelvis is identical in all three views for each of the three dyads.

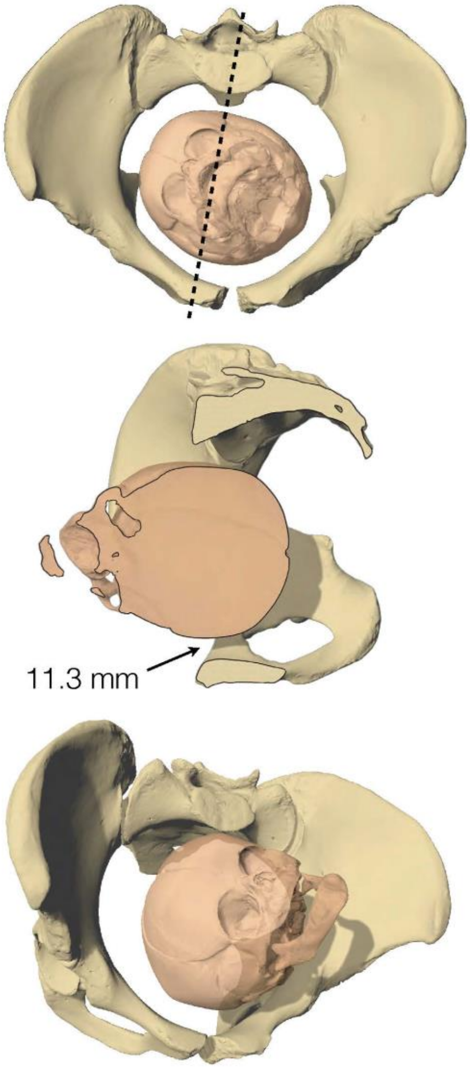

Supplementary Fig. 9. Engagement of the fetal head in the pelvic inlet of a modern human. The pelvis and the fetal skull are scaled to average dimensions (*see Methods*). This suggests that the soft tissue is compressed to a minimum thickness of 11.3 mm between the bony pelvic wall and the fetal skull if the head is centred within the birth canal. Top: view perpendicular to pelvic inlet, a dashed line indicates the plane where maximum constriction occurs. Middle: right lateral view, clipped at the plane of maximum constriction. Bottom: oblique perspective view. The orientation of the fetal head within the pelvis is identical in all three views.

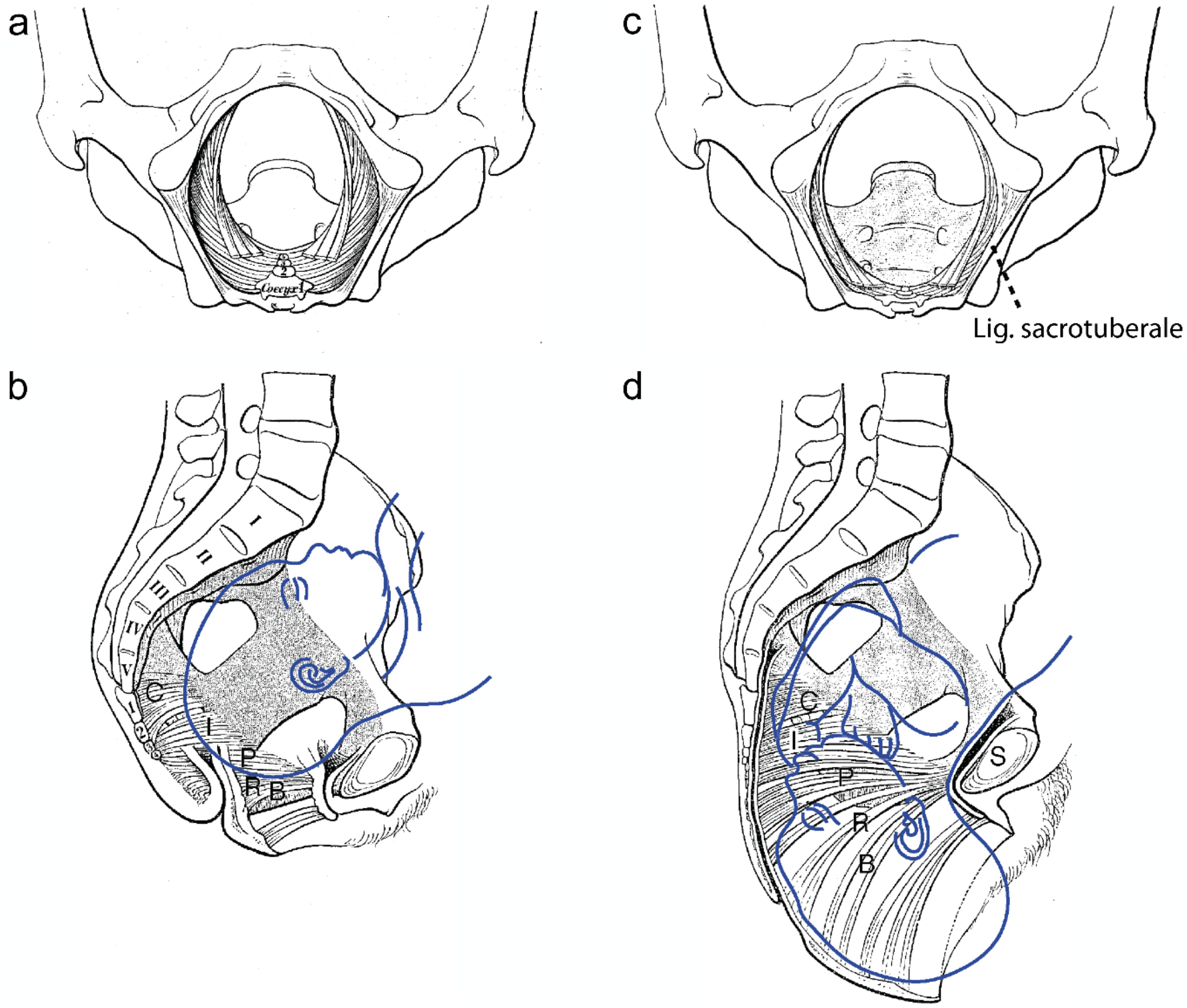

Supplementary Fig. 10. Schematic view of the muscular birth canal of a modern human female. **a,** perineal view, and **b,** median sagittal view of the pelvic diaphragm when the fetal head (blue outline) begins to stretch it. The fetal head is thereby guided into a sagittal orientation when entering the pelvic diaphragm, resulting in internal rotation. The levator hiatus has already lost its anteroposteriorly elongated slit shape to become elliptical before backward nutation of the sacrum and coccyx occurs. **c**, perineal view, and **d**, median sagittal view of the pelvic floor during maximum streching; the sacrum and coccyx are nutated backwards. In **a** and **c**, the praecoccygeal fibres of the puborectalis muscle are cut and all distal muscles have been removed to show the shape of the powerful muscular orifice. Abbreviations: C, m.coccygeus; I, m. ileococcygeus; P, m. pubococcygeus; R, m. puborectalis; B, m. bulbocavernosus; S, pubic symphysis with flattened bladder and urethra; the ileococcygeus, pubococcygeus and puborectalis mucles jointly form the levator ani. Please note that the fetal head becomes sagittally oriented despite the transversally oval bony pelvic outlet of this individual (transverse diameter 11 cm, anteroposterior diameter 8.5 cm, nutation of the sacrum and coccyx increases it to 11.5 cm). Measurements and drawings by L.H. Farabeuf, adapted from ref. 40, source: gallica.bnf.fr/BnF.

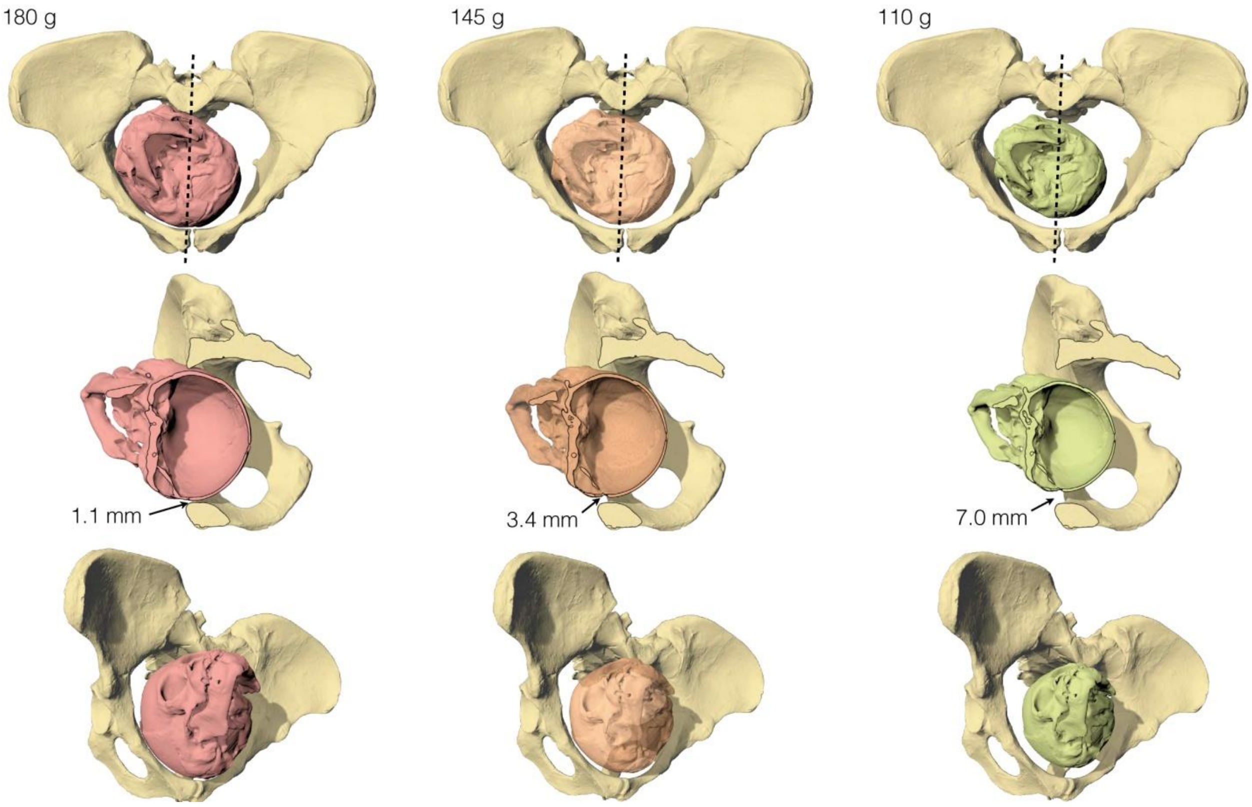

Supplementary Fig. 11. Engagement of a chimpanzee-based fetal head model in the pelvic inlet in A.L. 288-1, pelvic reconstruction of Häusler & Schmid[19]. The *in silico* simulation shows that only the 110 g fetal head size leaves sufficient space (i.e., > 7.0–10.6 mm) for fetopelvic soft tissue. The best cephalopelvic fit is obtained with a slightly oblique head presentation at the pelvic inlet, and the maximum constriction occurs in a para-sagittal plane (dashed line). Top row: view perpendicular to pelvic inlet. Middle row: right lateral view, clipped at the plane of maximum constriction; the figures indicate the width of the gap between the fetal skull and the maternal pelvis. Bottom row: oblique perspective view. The orientation of the fetal head within the pelvis is identical in all three views for each of the three dyads.

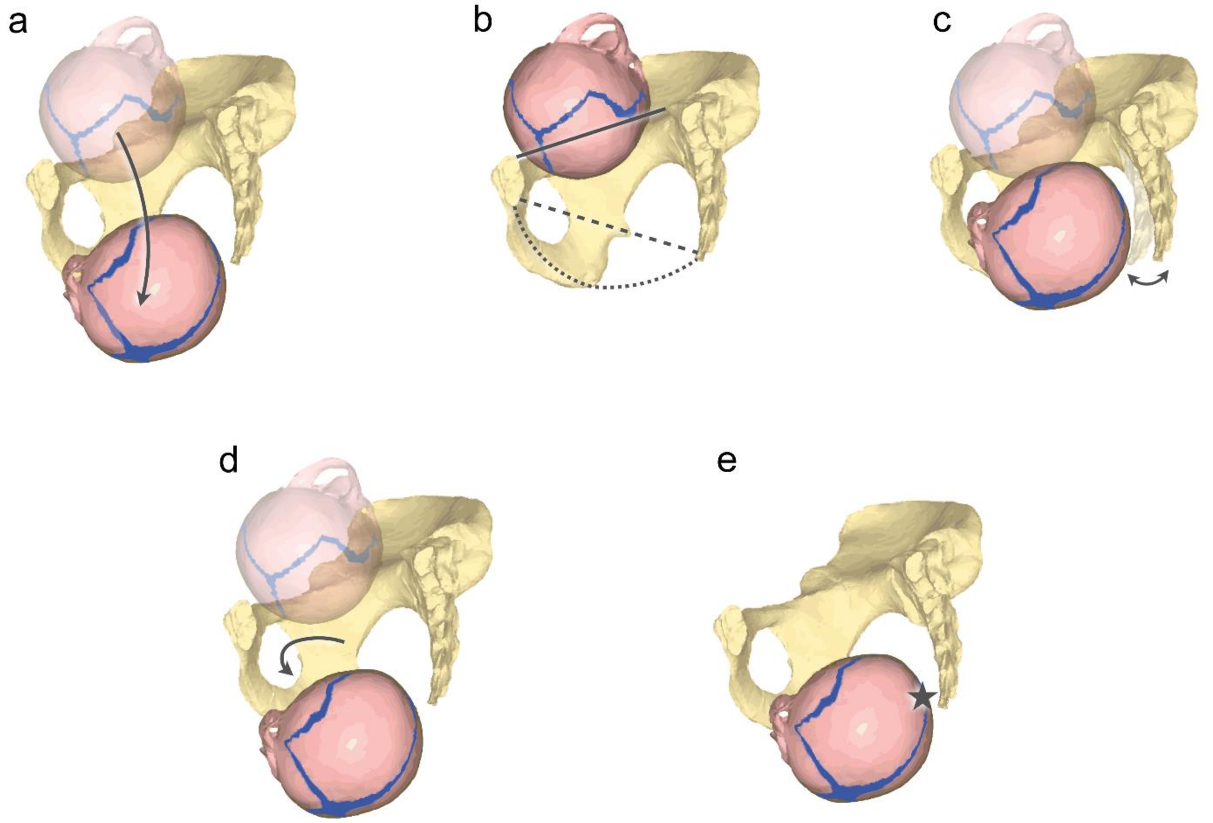

Supplementary Fig. 12. Obstetrical parameters. Lateral views of the maternal pelvic-fetal head dyads during bony birth simulations. The left hipbone has been removed for visualization purposes. **a**, eutocic birth: skull passing from the inlet (in transparent) through the entire pelvic canal. **b**, in dystocic birth simulations (arrest of descent of the skull), the level of arrest was noted (solid line: pelvic inlet; dashed line: midplane; dotted line: outlet. Please note that the inlet, midplane and outlet are actually 3D spaces as not all their points lie in the same plane, and that the bony outlet would extend to the tip of the coccyx[1]). **c**, the outlet can be enlarged during labour by outlet expansion, i.e., by backward rotation of the sacrum (nutation; double arrow). **d**, rotation of the skull (in degrees). **e,** position of the occiput (star) at the outlet. Note that internal rotation into the expected sagittal head orientation does not occur due to the absence of soft tissue in this model.

Supplementary Table 1. Adult and neonatal head size estimates based on adult-to-neonatal brain size proportions using different formulas

Species	Adult endocranial volume [cm^3]	Adult brain mass [grams] §	Neonatal brain mass using human relationship [grams] ‖	Neonatal brain mass using general primate formula [grams] #	Neonatal brain mass using catarrhine primate formula [grams] ¶
A. afarensis	445 (N=4) *	419	117	176	164
A. africanus	459 (N=8) †	432	121	184	168
A. sediba	420 (N=1) ‡	396	111	166	157

* based on A.L. 288-1 (dated to 3.18 Ma), A.L. 822-1 (dated to ~3.1 Ma), A.L. 333-45 (dated to ~3.2 Ma), A.L. 444-2 (dated to ~3.0 Ma)[23]

† based on MLD 1 (dated to 2.58–3.0 Ma), MLD 37/38 (dated to 2.58 Ma), Sts 5, Sts 19, Sts 60, Sts 71, StW 505 (all dated to 2.1–2.6 Ma), and Taung (dated to 2.58–3.0 Ma)[84]

‡ based on MH1 (dated to 1.98 Ma)[84]

§ Endocranial volume (C) can be transformed into brain weight (E) using the formula $C = 0.94 \times E^{1.02}$ (ref. [85])

‖ neonatal brain mass = 0.28 × adult brain mass [7], based on a mean neonatal brain mass of 368 g (N=79) and a mean adult brain mass of 1315 g (N = 864 men and 511 women aged 15 to 59 years) [77,78]

\# Log (neonatal brain mass) = 1.00 × Log (adult brain mass) – 0.37 (r = 0.992, N = 27) [7]

¶ Log (neonatal brain mass) = 0.7246 × Log (adult brain mass) + 0.3146 (r= 0.98, N = 7) [25]

Supplementary Table 2. Pelvic midplane and outlet dimensions as well as rotation of the fetal head derived from bony simulations (irrelevant for the actual birth outcome)

Pelvic reconstruction	Pelvic midplane AP [mm] *	Pelvic midplane TV [mm] *	Pelvic outlet AP [mm] *	Pelvic outlet TV [mm] *	Rotation in bony simulations †	Position at outlet in bony simulations ‡
A.L. 288-1 (Lovejoy et al.) [29]	70	106	73	95	45°/90°/45°	TR/–/–
A.L. 288-1 (Tague & Lovejoy) [18]	72	101	71	96	45°/45°/45°	TR/TR/-
A.L. 288-1 (Haeusler & Schmid) [19]	88	94	97	87	135°/45°/45°	OP/TR/–
A.L. 288-1 (Brassey et al.) [20]	103.6	86.7	114	76	0°/45°/0°	LOA/TR/–
Sts 14 (Haeusler & Schmid) [19]	86	89	85	107	-45°/45°/45°	OA/TR/–
Sts 14 (Berge & Goularas) [17]	73	93	-	105	135°/90°/45°	OP/LOP/–
MH2 (Kibii et al. and Laudicina et al.) [21,22]	97.9	-	97.4	-	90°/45°/45°	LOP/TR/–
Average modern human female§	121	113	119	120	45°	TR

* Published midplane and outlet dimensions may have been measured differently and may therefore not be exactly comparable. Please note that these dimensions significantly increase during labour due to outward rotation of the hipbones and nutation of the sacrum and their impact on birth outcome is therefore limited. In modern humans, the caudal tip of the sacrum can rotate backwards by 20–25 mm during labour[39,64]

† The direction of rotation is clockwise, starting from a left occiput anterior (LOA) position. The three values correspond to the bony simulations with a 180 g, 145 g, and 110 g fetal head size, respectively.

‡ Modern humans typically show an occiput anterior presentation at the outlet; deviations from this expected orientation are likely attributable to the absence of soft tissue in the bony simulations. The three positions at the outlet correspond to the bony simulations with a 180 g, 145 g, and 110 g fetal head size, respectively. TR: transverse head orientation at the outlet OP: Occiput Posterior; OA: Occiput Anterior; LOP: Left Occiput Posterior.

§ Based on 15 reproductive-aged females of the Weisbach collection (Natural History Museum Vienna)

Supplementary Table 3. Neonatal head dimensions

Neonatal brain size [grams]	Neonatal head length [mm]	Neonatal biparietal head diameter [mm]
110	75	64
145	81	70
155*	83	71
180	87	75
368	111	90

* corresponding to an average chimpanzee newborn; only used in the *in silico* simulations
