# Supplementary material for: Dynamic finite-element simulations reveal early origin of complex human birth pattern
Source: Commun Biol. 2022 Apr 19;5:377. doi: 10.1038/s42003-022-03321-z (PMC9018746; doi:10.1038/s42003-022-03321-z)
Supplement: Supplementary file 2 — Reporting Summary [file 42003_2022_3321_MOESM2_ESM.pdf]

nature research

Corresponding author(s): Frémondière Pierre

Last updated by author(s): Mar 9, 2022

Reporting Summary

Nature Research wishes to improve the reproducibility of the work that we publish. This form provides structure for consistency and transparency in reporting. For further information on Nature Research policies, see our Editorial Policies and the Editorial Policy Checklist.

Statistics

For all statistical analyses, confirm that the following items are present in the figure legend, table legend, main text, or Methods section.

n/a	Confirmed	
☐	☒	The exact sample size (*n*) for each experimental group/condition, given as a discrete number and unit of measurement
☒	☐	A statement on whether measurements were taken from distinct samples or whether the same sample was measured repeatedly
☒	☐	The statistical test(s) used AND whether they are one- or two-sided<br>*Only common tests should be described solely by name; describe more complex techniques in the Methods section.*
☒	☐	A description of all covariates tested
☒	☐	A description of any assumptions or corrections, such as tests of normality and adjustment for multiple comparisons
☒	☐	A full description of the statistical parameters including central tendency (e.g. means) or other basic estimates (e.g. regression coefficient) AND variation (e.g. standard deviation) or associated estimates of uncertainty (e.g. confidence intervals)
☒	☐	For null hypothesis testing, the test statistic (e.g. *F*, *t*, *r*) with confidence intervals, effect sizes, degrees of freedom and *P* value noted<br>*Give P values as exact values whenever suitable.*
☒	☐	For Bayesian analysis, information on the choice of priors and Markov chain Monte Carlo settings
☒	☐	For hierarchical and complex designs, identification of the appropriate level for tests and full reporting of outcomes
☒	☐	Estimates of effect sizes (e.g. Cohen's *d*, Pearson's *r*), indicating how they were calculated

Our web collection on statistics for biologists contains articles on many of the points above.

Software and code

Policy information about availability of computer code

Data collection	Pelvic meshes were obtained from different authors (O.Lovejoy, D. Goularas, P. Schmid and N. Laudicina provided their reconstruction of AL288-1, Sts-14 and MH2, respectively). We then used Geomagic (www.3dsystems.com) to isolate the sacrum and Hypermesh 12.0 (www.altair.com) to generate new models with a simplified geometry. We used Mimics 12.3 (www.materialise.com) to generate fetal head meshes based on medical data
Data analysis	The birth simulations were performed with finite-element analyses (FEA) using the commercial software Radioss 11.0 Radioss is a solver of FEA that belong to the hyperworks environment (www.altair.com)<br>In silico "simulations" were performed with Rhinoceros 7.0 (www.rhino3d.com)

For manuscripts utilizing custom algorithms or software that are central to the research but not yet described in published literature, software must be made available to editors and reviewers. We strongly encourage code deposition in a community repository (e.g. GitHub). See the Nature Research guidelines for submitting code & software for further information.

Data

Policy information about availability of data

All manuscripts must include a data availability statement. This statement should provide the following information, where applicable:

- Accession codes, unique identifiers, or web links for publicly available datasets
- A list of figures that have associated raw data
- A description of any restrictions on data availability

All data are available in the main text or the supplementary materials. Correspondence and requests for materials should be addressed to M.H. and P.F.

Field-specific reporting

Please select the one below that is the best fit for your research. If you are not sure, read the appropriate sections before making your selection.

☐ Life sciences ☐ Behavioural & social sciences ☒ Ecological, evolutionary & environmental sciences

For a reference copy of the document with all sections, see nature.com/documents/nr-reporting-summary-flat.pdf

Ecological, evolutionary & environmental sciences study design

All studies must disclose on these points even when the disclosure is negative.

Study description	Here, we explore the obstetrical dilemma with finite-element birth simulations in Australopithecus using different fetal head sizes. We show that adaptation to bipedalism and the corresponding reshaping of the pelvis in these early hominins led to a tight fit between the mother's pelvis and the newborn head despite their relatively small brain sizes. To relieve this dilemma, australopithecines must have already given birth to secondarily altricial infants that were neurologically less developed than great apes. The evolution of a modern pattern of life history and cognitive development therefore seems to have predated the appearance of the genus Homo.
Research sample	We used 3 female australopithecine fossils preserve enough of the hipbones and sacrum to allow a reliable reconstruction of the pelvis, including A.L. 288-1 (Australopithecus afarensis), dated to 3.18 millions years ago (Ma) (4 reconstructions), Sts 14 (A. africanus, 2.6-2.1 Ma) (2 reconstructions) and MH2 (A. sediba, 1.98 Ma) (1 reconstruction). All these specimens are of similarly small body size. In combination with these pelvises, we used 3 fetal head meshes scaled to conform to the brain masses of 180g-145g-110g using neurocranial dimensions of a chimpanzee neonate. This generates 21 virtual mother-infant dyads.
Sampling strategy	Since there is not enough pelvic reconstructions, we did not choose any sampling strategy: we used all available pelvic reconstructions in the litterature
Data collection	The manual reconstruction of A.L. 288-1 and Sts-14 by Hausler and Schmid and the reconstruction of MH2 by Kibii et al. were scanned with a high-resolution surface scanner (PT-M4c, Polymetric GmbH, Darmstadt, Germany), while the other reconstructions were provided by the corresponding authors as digital models (Martin Hausler, Cinzia Fornai, Nicole Webb). Our fetal skull model was based on CT scan of a human fetus at 35 weeks of gestation. The CT scan was performed with a 16 slice Siemens SOMATOM Definition Flash strip scanner with 0.6 mm slice thickness. The CT images were segmented in Mimics 12.3. the generated polygonal mesh of the fetal head was re-meshed in Hypermesh 12.0 to produce 18000 shell elements with an average size of 1 mm (Pierre Frémondière, François Marchal, Lionel Thollon).
Timing and spatial scale	We collected the pelvic reconstruction of AL- 288-1 by CO Lovejoythe 24 May 2018<br>We had the reconstruction of Sts-14 of D Goularas the 4 june 2019<br>We obtained the reconstruction of MH2 by N Laudicina the 16 october 2019<br>The other reconstructions were already in our possession<br>Time between data acquisition is explained by the multiple simulations we performed as initial steps of the research work. The fetal CT scan was performed the 1 october 2012 (1d-RCB 2011-A00072-39). Meshes were generated between April and december 2013, during the PhD of P Frémondière.
Data exclusions	They were no exclusion criteria
Reproducibility	the pelvic models were checked and compared to the published measurements (inlet antero-posterior, transverse, bispinous, biischiatic)
Randomization	Given the low number of pelvises, we did not allocated pelvises into different study groups
Blinding	Given the low number of pelvises, blinding was not relevant for the data acquisition

Did the study involve field work? ☐ Yes ☒ No

Reporting for specific materials, systems and methods

We require information from authors about some types of materials, experimental systems and methods used in many studies. Here, indicate whether each material, system or method listed is relevant to your study. If you are not sure if a list item applies to your research, read the appropriate section before selecting a response.

Materials & experimental systems

n/a	Involved in the study	
☒	☐	Antibodies
☒	☐	Eukaryotic cell lines
☐	☒	Palaeontology and archaeology
☒	☐	Animals and other organisms
☒	☐	Human research participants
☒	☐	Clinical data
☒	☐	Dual use research of concern

Methods

n/a	Involved in the study	
☒	☐	ChIP-seq
☒	☐	Flow cytometry
☒	☐	MRI-based neuroimaging

Palaeontology and Archaeology

Specimen provenance	AL 288-1 is from Hadar, Ethiopia, discovered in 1974.<br>Sts-14 is from Sterkfontein, South-africa, discovered in 1947.<br>Mh2 is from Malapa, South-africa, discovered in 2008.<br>Since these pelvic reconstructions were already published, no permits were obtained to work with these specimens
Specimen deposition	Surface scans of pelvises were kindly provided by the different authors of these reconstructions and transmitted to Martin Hausler
Dating methods	We did not change dates attributed to these specimens

☒ Tick this box to confirm that the raw and calibrated dates are available in the paper or in Supplementary Information.

Ethics oversight	Concerning the use of pelvic reconstructions, no ethical approval was required since it did not involve human participation.<br>Concerning the use a CT scan of the human fetus, this data was collected in the frame of a clinical study (PhD of P Frémondière) and was authorized after an ethical board validation (1d-RCB 2011-A00072-39).

Note that full information on the approval of the study protocol must also be provided in the manuscript.
